# Supplementary material for: Hoxa2 Selectively Enhances Meis Binding to Change a Branchial Arch Ground State
Source: Dev Cell. 2015 Feb 9;32(3):265–77. doi: 10.1016/j.devcel.2014.12.024 (PMC4333904; doi:10.1016/j.devcel.2014.12.024)
Supplement: Document S1. Supplemental Experimental Procedures, Figures S1–S5, and Table S5 [file mmc1.pdf]

Developmental Cell

Supplemental Information

Hoxa2 Selectively Enhances Meis Binding to Change a Branchial Arch Ground State

Shilu Amin, Ian J. Donaldson, Denise A. Zannino, James Hensman, Magnus Rattray, Marta Losa, François Spitz, Franck Ladam, Charles G. Sagerström, and Nicoletta Bobola

Supplemental Figures

Figure S1

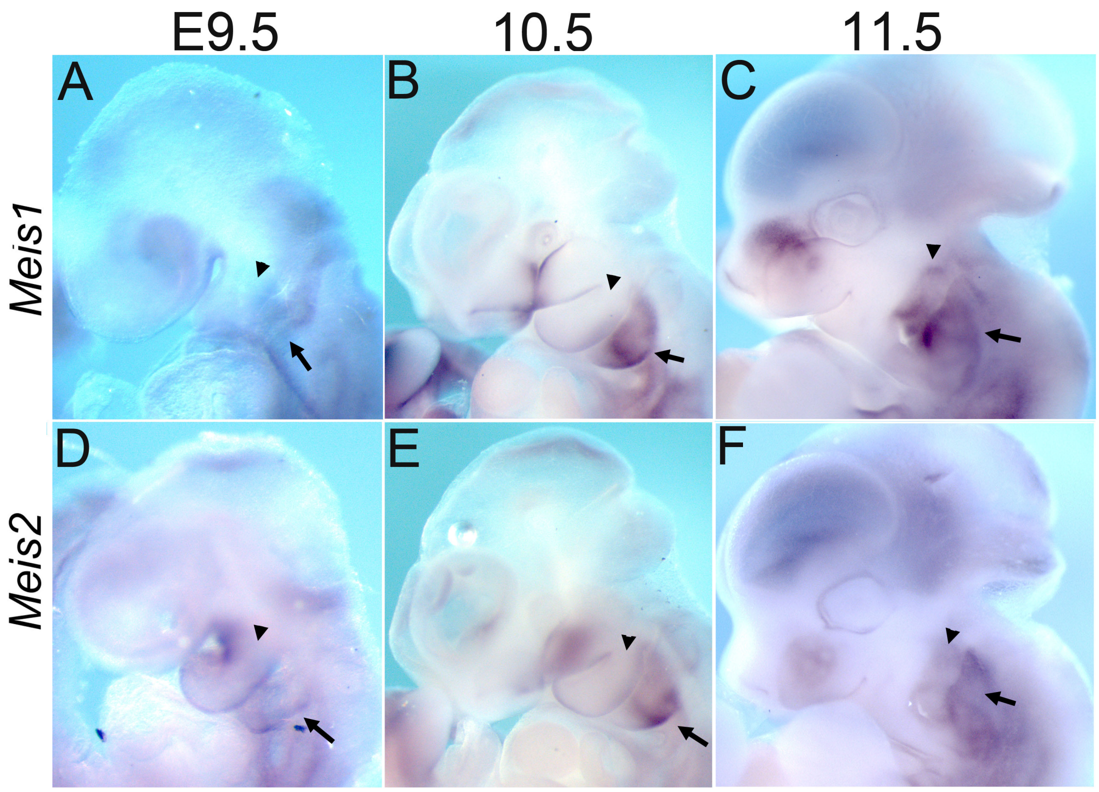

Figure S2

A

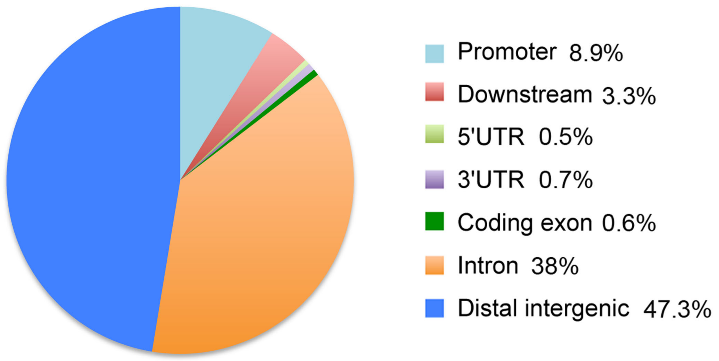
Promoter 8.9%
Downstream 3.3%
5'UTR 0.5%
3'UTR 0.7%
Coding exon 0.6%
Intron 38%
Distal intergenic 47.3%

B

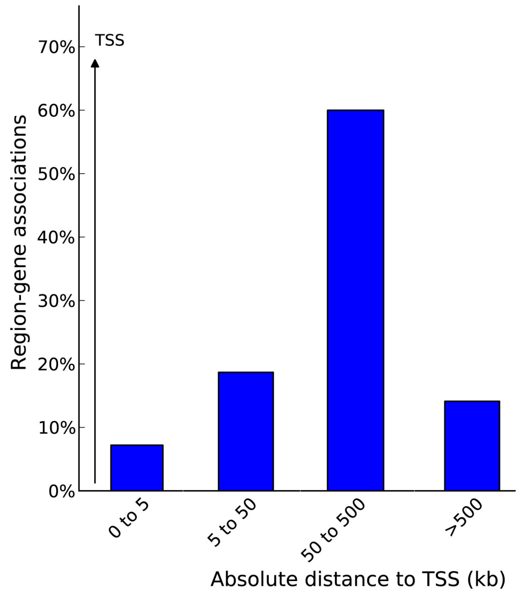
TSS
Region-gene associations
70%
60%
50%
40%
30%
20%
10%
0%
0 to 5
5 to 50
50 to 500
>500
Absolute distance to TSS (kb)

Figure S3

A

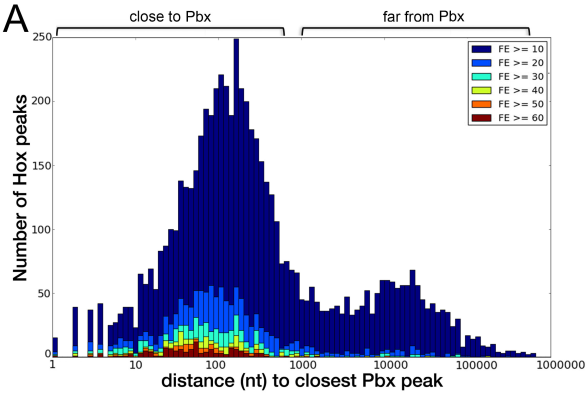

B

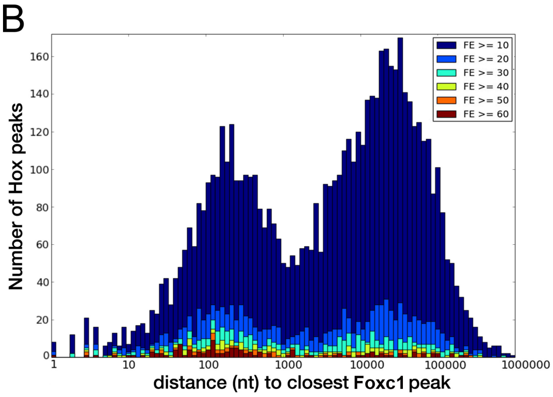

C

	Hoxa2	Meis	Pbx	Foxc1
**Hoxa2**	10031	5116	4688	1852
**Meis**		62627	31603	8015
**Pbx**			59341	7083
**Foxc1**				30384

D

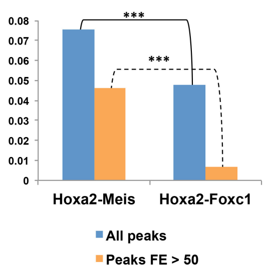

E

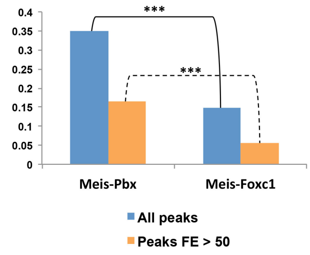

Figure S4

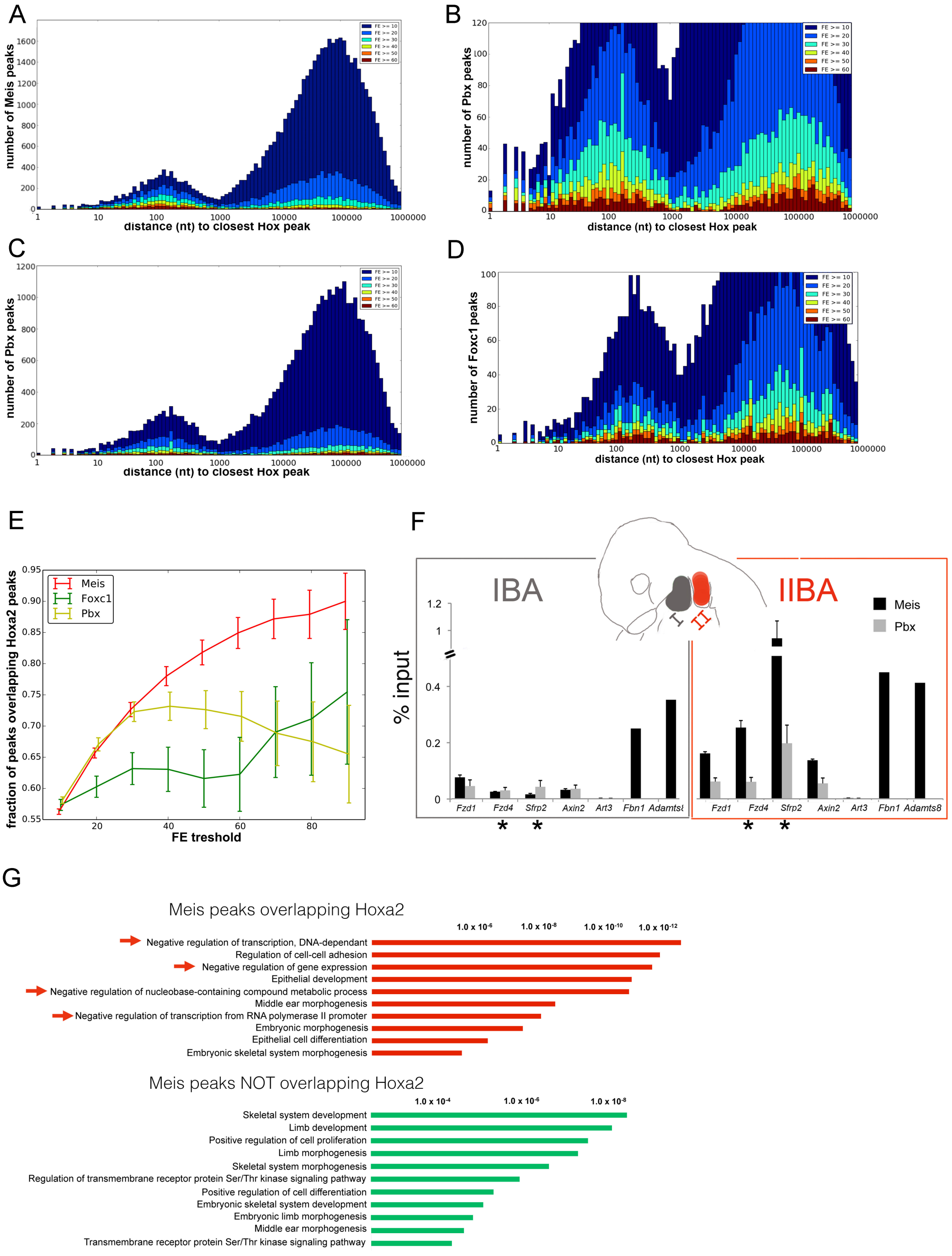
A
number of Meis peaks
distance (nt) to closest Hox peak
FE >= 10
FE >= 20
FE >= 30
FE >= 40
FE >= 50
FE >= 60
B
number of Pbx peaks
distance (nt) to closest Hox peak
C
number of Pbx peaks
distance (nt) to closest Hox peak
D
number of Foxc1 peaks
distance (nt) to closest Hox peak
E
Meis
Foxc1
Pbx
fraction of peaks overlapping Hoxa2 peaks
FE treshold
F
IBA
IIBA
Meis
Pbx
% input
Fzd1
Fzd4
Sfrp2
Axin2
Art3
Fbn1
Adamts8
G
Meis peaks overlapping Hoxa2
Negative regulation of transcription, DNA-dependant
Regulation of cell-cell adhesion
Negative regulation of gene expression
Epithelial development
Negative regulation of nucleobase-containing compound metabolic process
Middle ear morphogenesis
Negative regulation of transcription from RNA polymerase II promoter
Embryonic morphogenesis
Epithelial cell differentiation
Embryonic skeletal system morphogenesis
Meis peaks NOT overlapping Hoxa2
Skeletal system development
Limb development
Positive regulation of cell proliferation
Limb morphogenesis
Skeletal system morphogenesis
Regulation of transmembrane receptor protein Ser/Thr kinase signaling pathway
Positive regulation of cell differentiation
Embryonic skeletal system development
Embryonic limb morphogenesis
Middle ear morphogenesis
Transmembrane receptor protein Ser/Thr kinase signaling pathway

Figure S5

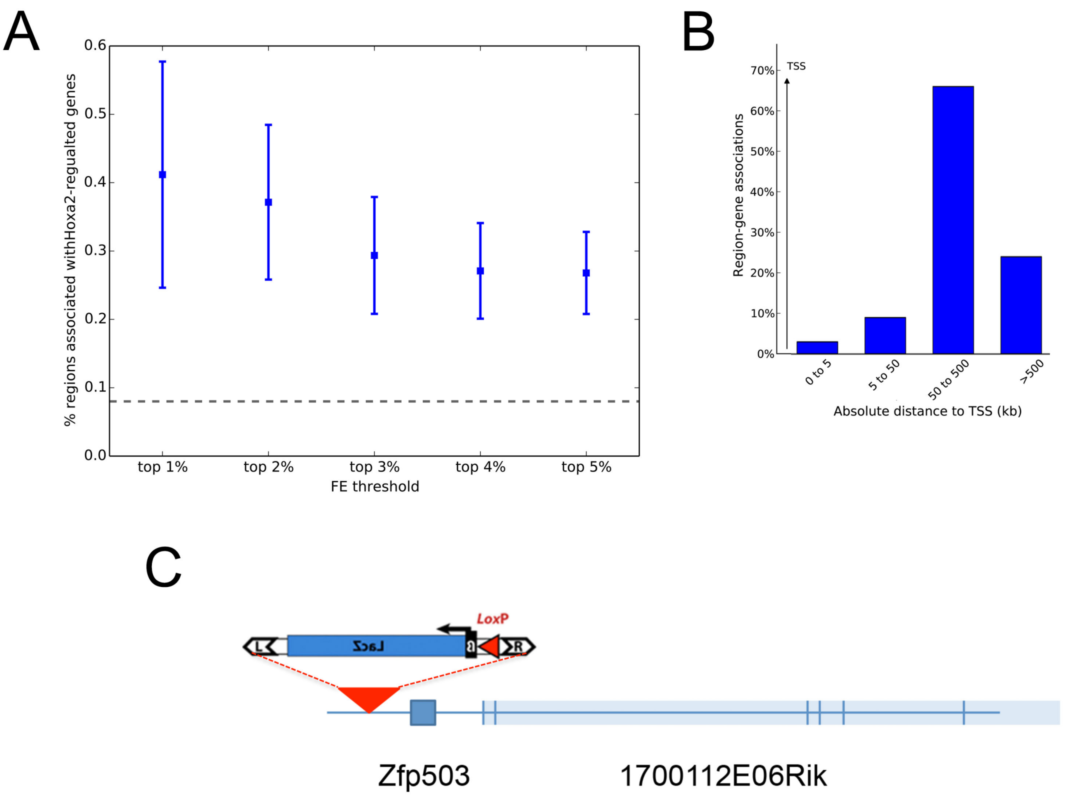
A
0.6
0.5
0.4
0.3
0.2
0.1
0.0
% regions associated withHoxa2-regualted genes
top 1%
top 2%
top 3%
top 4%
top 5%
FE threshold
B
70%
60%
50%
40%
30%
20%
10%
0%
TSS
Region-gene associations
0 to 5
5 to 50
50 to 500
>500
Absolute distance to TSS (kb)
C
LoxP
L
LacZ
R
Zfp503
1700112E06Rik

Supplemental Figures legends

Figure S1 (related to Figure 1). Expression of *Meis1* and *Meis2* in the craniofacial region. Whole-mount ISH on wild-type E9.5 (A, D), E10.5 (B, E) and late E11.5 (C, F) mouse embryos, using *Meis1* (A-C) and *Meis2* (D-F) probes. The IIBA (arrows) is one of the main domains of expression of *Meis1* and *Meis2*. Arrowheads indicate the first arch.

Figure S2 (related to Figure 2). **Genome-wide occupancy of Meis TFs in the IIBA**. Distribution of Meis summit regions relative to Reference Sequence (RefSeq) gene structures. The pie charts indicate the proportion of reads for each gene structure; the corresponding numeric values are included. B. Distribution of Meis summits relative to the absolute distance to a trancription start site (TSS).

Figure S3 (related to Figure 4). Combinatorial binding of Hoxa2, Meis, Pbx and Foxc1 A. Distance of Hoxa2 peaks (IIBA) relative to Pbx peaks. Hoxa2 peaks are binned according to distance to the nearest Pbx peak and labeled according to fold enrichment (FE; high FE is represented by dark red bars and low FE is represented by dark blue bars). B. Distance of Hoxa2 peaks relative to Foxc1 peaks. Hoxa2 peaks are binned according to distance to a nearest Foxc1 peak and labeled according to FE, as above. C. Total number of peaks for Hoxa2, Meis, Pbx and Foxc1 followed by pairwise combinations (number of overlapping 200nt summits). D, E. Significance of pairwise overlaps The co-binding frequency of Hoxa2/Meis is significantly higher than Hoxa2/Foxc1 (D). The co-binding frequency of Meis/Pbx is significantly higher than Meis/Foxc1 (E). Using a binomial test, similar results were observed using the entire ChIP-seq (blue bars) and peaks with FE>50 (orange bars). Asteriks indicate a P value <1E-8.

Figure S4 (related to Figure 5). Hoxa2 enhances Meis binding. A. Distance of Meis peaks (IIBA) relative to Hoxa2 peaks (non cropped). Meis peaks are binned according to distance to the nearest Hoxa2 peak and labeled according to fold enrichment (FE; high FE is represented by dark red bars and low FE is represented by dark blue bars). B, C. Distance of Pbx peaks relative to Hox peaks, cropped (B) and uncropped (C). Pbx peaks are binned according to distance to a nearest Hoxa2 peak and labeled according to FE, as above. D. Distance of Foxc1 peaks relative to Hox peaks (cropped). Foxc1 peaks are binned according to distance to a nearest Hoxa2 peak and labeled according to FE, as above. E. Distribution of Meis, Pbx and Foxc1 peaks relative to Hoxa2 binding. The fraction of Meis (red), Pbx (yellow) and Foxc1 (green) peaks close (1kb) to a Hoxa2 peak are plotted with error bars showing 95% confidence region. High Meis peaks (FE≥40) occur at significantly higher frequencies close to Hoxa2 (1kb) than Pbx or Foxc1 peaks. F. Meis and Pbx occupancy in the IBA (grey panel) and IIBA (red panel) by ChIP-qPCR. *Fzd1*, *Fzd4*, *Sfrp2* and *Axin2* are Hoxa2-bound regions; the IIBA/IBA average enrichment ratios at these regions were 10 and 2.41 for Meis and Pbx, respectively. Regions bound by Meis alone (i.e., *Fbn1, Adamts8*, closest Hoxa2 binding >10 kb) showed comparable enrichments when chromatin was extracted from the IBA or the IIBA. *Art3* is a negative control (unbound region). Asterisks indicate regions associated with Hoxa2-regulated genes. Data are presented as average of two independent experiments in duplicate and error bars represent the standard error of the mean. G. Functional categories associated with the top Meis peaks in the IIBA that overlap (red) or do not overlap (green) a Hoxa2 peak (200nt peak summit regions). Meis peaks overlapping Hoxa2 binding display enrichment in the GO term 'negative regulation of transcription' (red arrows), similar to the entire set of top Meis peaks in the IIBA. The length of the bars corresponds to the binomial raw (uncorrected) P-values (x-axis values).

Figure S5 (Related To Figure 6). Hoxa2 and Meis synergistic binding. A. Association of Hoxa2/Meis synergistic binding regions with Hoxa2-regulated genes. Increasing the number of 'synergistic binding events' (by relaxing the FE threshold) results in lower, but still significant associations with Hoxa2-regulated genes, relative to the entire Hoxa2 ChIP-seq (dashed horizontal line at 8%). Top1% (x-axis) indicates the set of top 1% Meis peaks that overlap top1% Hoxa2 peaks; top 2% indicates the set of top 2% Meis peaks that overlap top 2% Hoxa2 peaks, etc. The number of overlapping binding events are : top1%: n= 34; top 2%: n = 70; top 3%: n = 109; top 4%: n = 155; top 5%: n = 209. B. Distribution of Hoxa2/Meis synergistic binding regions relative to the transcription start site (TSS). C. Transposon insertion in the genomic regions containing *Zfp503*. The insertion is located downstream of *Zfp503* (red triangle).

Supplemental Tables and Legends

Table S1 (related to Figure 2). Meis ChIP-seq dataset in the IIBA

Table S2 (related to Figure 2). Meis ChIP-seq dataset in the IBA

Table S3 (related to Figure 2). Pbx ChIP-seq dataset in the IIBA

Table S4 (related to Figure 5). **Hoxa2-Meis synergistic binding regions.** List of high confidence Hoxa2-Meis cooperative binding, their associated genes and occurrence of Hox and Meis motifs. Genes dysregulated in *Hoxa2* mutant embryos are in bold.

Table S5 (related to Experimental procedure). List of primers used in ChIP-qPCR

**Gene**	**Forwards primer**	**Reverse primer**
Meis1 (1) enhancer	AGATGCCCAGAGAAAGCAAA	GGGGTGTGCATAGAAGGAAA
Meis1 (2) transcript	GCCAATCTTAGCGTCCATTT	TCACTCCGCCATTCTAAACA
Meis2 (1) enhancer	GGGGCGATGCTGTTTATTTA	GGGTGACAGGAATGATGGAT
Meis2 (2) promoter	TCCCGCAGACATCATTGTTA	AAATTTGGCCTCGTTCTCCT
Pou6f2 (Donaldson et al. 2012)	CCTGGATTTGACTCGGAAAG	CAGCTGTAACCCGACATTATGA
Itih4 neg (Donaldson et al. 2012)	GAGTCTGCTTGGCTTGAACC	AAGATTGGGCACTTTTTGGA
Hoxa2	TGAGGCGTTCCTTTCTGACT	GGGACCGCGCTACTATTAAA
Fzd1 (Donaldson et al. 2012)	GCCAGAAAATTGTTTGCATAAT	AGAGGGCTGTGGAATTCTGA
Fzd4 (Donaldson et al. 2012)	CTTCCTGGCTCTGCTGAAAG	CAAGGACTCCCATTTACCTCA
Sfrp2 (Donaldson et al. 2012)	TCACCATGACAGGTGGAAAA	CGGAGCTGAAACAGAAGCTC
Axin2 (Donaldson et al. 2012)	TGCTAAAAAGCCGGGAAGTA	TCGCAGTCCCTTTGAACTCT
Art3 neg (Donaldson et al. 2012)	GCCAAATTTAGCCAGATCCA	ACGGGGCAACAATTAGCTTT
Fbn1	CCTACCAGCCTGTGTCAATT	TCCTTTTCCCAAGTCTCCAGT
Adamts8	CAGCCTCCACTTAGCCAATT	GGCAACATTTCTGGCACTTTC

Zfp503	TCCTGGTCTGTTTAATGTTTGCT	GCCATCATCTAAAGCACAGAGG
Zfp703	GGCTCCGATGGCTGTAATAA	TGCATTTGCAAAGACGGCTA
Wnt5a	AAGACCTCCTTGCGATCTGA	AAACACCAGGGGCAATCATA
Cxcl12	CACTGATTGGAACCAGATGGG	GCTTGCCAAGTGTGTCGCTAA
Neg Chr17	TGAAGTACAGAAGCGAATGAAGG	TTTGCATGCCAGTGATACTTATC

Supplemental Experimental procedures

ISH probes

Probes used for *in situ* hybridization were *Meis1*, *Zfp503* (gifts from Dorothea Schulte and Carol Wicking), *Meis2* (targeting Meis2 3'UTR), *Zfp703* (Slorach et al., 2011) for mouse and *dlx2a* (Akimenko et al., 1994) and *hoxa2b* (Prince and Lumsden, 1994; Prince et al., 1998) for zebrafish.

Antisense morpholino oligonucleotides injections

Antisense morpholino (MO) oligonucleotides were designed to the translation start sites of *meis1* (5'- TATCTTCGTACCTCTGCGCCATCGC-3'), *meis2a* (5'-CTCATCGTACCTTTGCGCCATCAGC -3'), *meis3* (5'-AACTCCTCATACCTCTTATCCATGC-3'), and *meis4.1a* (5'-AGATCCTCGTACCGTTGCGCCATGA-3'), with a corresponding 5bp mismatch MO designed to *meis4.1a* (5'- AGtTgCTCcTACCcTTcCGCCATGA-3') (Gene Tools, LLC). A combination of four antisense morpholinos (MOs) was injected, each with a concentration of 0.7 mM (final combined concentration of 2.8 mM) in 1nl total volume. A corresponding 2.8 mM mismatch control MO was also used.
