# Supplementary material for: Hoxa2 Selectively Enhances Meis Binding to Change a Branchial Arch Ground State
Source: Dev Cell. 2015 Feb 9;32(3):265–77. doi: 10.1016/j.devcel.2014.12.024 (PMC4333904; doi:10.1016/j.devcel.2014.12.024)
Supplement: Document S2. Article plus Supplemental Information [file mmc6.pdf]

Article

Developmental Cell

Hoxa2 Selectively Enhances Meis Binding to Change a Branchial Arch Ground State

Highlights

- Meis provides a ground state that is common to all the branchial arches
- Hoxa2 recognizes Meis prebound sites in the second arch that contain Hox motifs
- Hoxa2 enhances Meis binding, which coincides with active enhancers, at these sites
- Hoxa2 modulates the ground-state binding of Meis to instruct second arch identity

Authors

Shilu Amin, Ian J. Donaldson, ..., Charles Sagerström, Nicoletta Bobola

Correspondence

nicoletta.bobola@manchester.ac.uk

In Brief

Hox transcription factors instruct specific morphologies in the branchial arches, a series of segments in the developing vertebrate head. Amin et al. found that Hoxa2 operates as a tissue-specific cofactor, enhancing binding of the homeodomain transcription factor Meis to specific sites that provide the second arch with its anatomical identity.

CrossMark

Amin et al., 2015, Developmental Cell *32*, 265–277
February 9, 2015 ©2015 The Authors
http://dx.doi.org/10.1016/j.devcel.2014.12.024

CellPress

Developmental Cell
Article

CellPress

Hoxa2 Selectively Enhances Meis Binding to Change a Branchial Arch Ground State

Shilu Amin,[1] Ian J. Donaldson,[2] Denise A. Zannino,[3] James Hensman,[4,5] Magnus Rattray,[2] Marta Losa,[1] François Spitz,[6] Franck Ladam,[3] Charles Sagerström,[3] and Nicoletta Bobola[1,7,*]
[1]School of Dentistry, Faculty of Medical and Human Sciences, University of Manchester, Manchester M13 9PT, UK
[2]Faculty of Life Sciences, University of Manchester, Manchester M13 9PT, UK
[3]Department of Biochemistry and Molecular Pharmacology, University of Massachusetts Medical School, 364 Plantation Street, Worcester, MA 01655, USA
[4]Department of Computer Science, University of Sheffield, Sheffield S1 4DP, UK
[5]The Sheffield Institute for Translational Neuroscience, Sheffield S10 2HQ, UK
[6]Developmental Biology Unit, European Molecular Biology Laboratory, 69117 Heidelberg, Germany
[7]Institute of Human Development, Faculty of Medical and Human Sciences, Manchester Academic Health Science Centre, University of Manchester, Manchester M13 9PT, UK
*Correspondence: nicoletta.bobola@manchester.ac.uk
http://dx.doi.org/10.1016/j.devcel.2014.12.024
This is an open access article under the CC BY license (http://creativecommons.org/licenses/by/4.0/).

SUMMARY

Hox transcription factors (TFs) are essential for vertebrate development, but how these evolutionary conserved proteins function in vivo remains unclear. Because Hox proteins have notoriously low binding specificity, they are believed to bind with cofactors, mainly homeodomain TFs Pbx and Meis, to select their specific targets. We mapped binding of Meis, Pbx, and Hoxa2 in the branchial arches, a series of segments in the developing vertebrate head. Meis occupancy is largely similar in Hox-positive and -negative arches. Hoxa2, which specifies second arch (IIBA) identity, recognizes a subset of Meis prebound sites that contain Hox motifs. Importantly, at these sites Meis binding is strongly increased. This enhanced Meis binding coincides with active enhancers, which are linked to genes highly expressed in the IIBA and regulated by Hoxa2. These findings show that Hoxa2 operates as a tissue-specific cofactor, enhancing Meis binding to specific sites that provide the IIBA with its anatomical identity.

INTRODUCTION

The body plan of vertebrates involves the formation of similar repetitive segments, which subsequently diversify to give rise to different body parts. A key discovery has been that Hox genes endow the initially identical segments with their distinct identities (McGinnis et al., 1984a, 1984b; Scott and Weiner, 1984).

Branchial arches are a useful model of segmental specification. This transient, metameric series of tissue bands appears in the head region of vertebrate embryos. Cranial neural crest (CNC) cells, emerging from areas of the hindbrain that express different Hox genes, colonize the branchial arches (Le Douarin and Kalcheim, 1999). All branchial arches share a ground-patterning program that is fully executed in the Hox-free first branchial arch. Hox proteins reprogram the execution of this first arch-like program in the subsequent arches. Hoxa2, a member of the Hox paralog group 2, patterns the second arch skeleton. In embryos that lack Hoxa2 function, the second branchial arch (IIBA) forms mirror image copies of first arch skeletal derivatives (Gendron-Maguire et al., 1993; Rijli et al., 1993). In addition, simultaneous inactivation of all HoxA cluster genes in the CNC leads to partial replacement of second, third, and fourth arch skeletal derivatives with multiple first arch-like structures (Minoux et al., 2009). Thus, Hox proteins appear to instruct arch-specific morphologies by overriding a ground-patterning program. Accordingly, in IIBA territory, Hoxa2 represses the expression of genes that are expressed in the anterior first branchial arch (IBA) (Bobola et al., 2003; Geisen et al., 2008; Kirilenko et al., 2011; Kutejova et al., 2005, 2008).

Intensive work in the past years has revealed that Hox genes are present in nearly all animals, and the principles of Hox gene organization and function are highly conserved throughout the animal kingdom (Carroll, 1995; Krumlauf, 1994; Lemons and McGinnis, 2006; Noordermeer and Duboule, 2013; Pearson et al., 2005; Trainor and Krumlauf, 2001). Despite this enormous progress, the logic of segment specification remains difficult to grasp.

As DNA-binding transcription factors, Hox display notoriously low binding specificity in vitro, yet they accomplish the task of selecting specific target genes to control segment morphology (Berger et al., 2008; Mann et al., 2009; Noyes et al., 2008). Hox proteins bind cooperatively with three amino acid loop extension (TALE) homeodomain transcription factors Pbx and Meis (Mann et al., 2009). Because complex formation improves the affinity and sequence selectivity of Hox proteins, the prevailing view has been that Pbx and Meis act as ancillary cofactors and assist Hox proteins in identifying their specific targets in the genome (Mann et al., 2009; Moens and Selleri, 2006). The main limitation of this view is that it is largely based on molecular and biochemical analyses in vitro. Insight into the functional interactions of Hox and TALE cofactors in their native environment in the embryo remains limited, in large part because these interactions have yet to be mapped on a genome-wide scale.

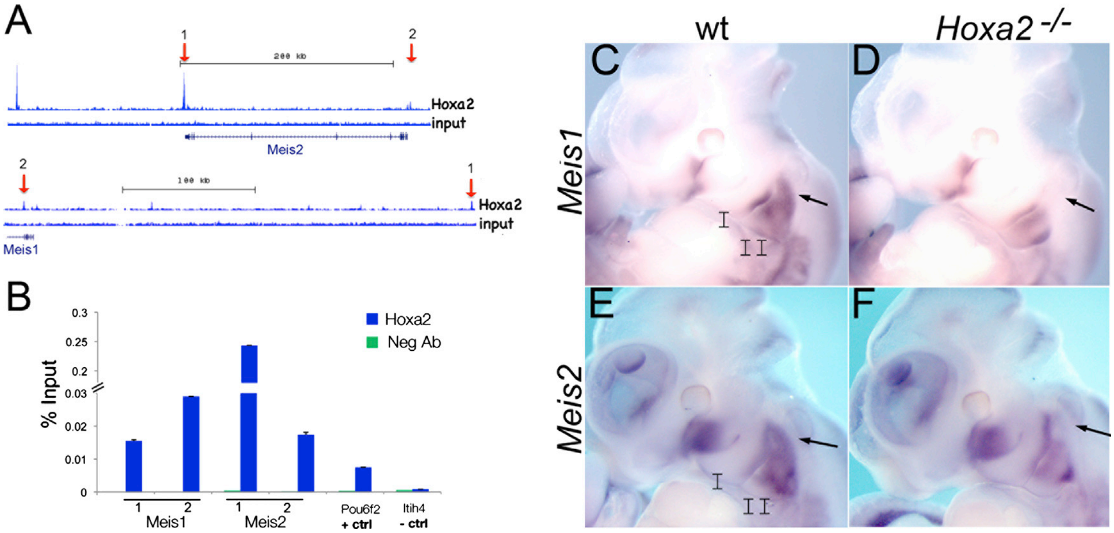

Figure 1. Hoxa2 Activates *Meis1* and *Meis2* in the IIBA

(A) ChIP-seq-binding profile of Hoxa2 at *Meis1* and *Meis2* genes in E11.5 IIBA. Input tracks represent control genomic DNA. Arrows highlight the binding regions tested by ChIP qPCR in (B).

(B) Hoxa2 binding to *Meis1* and *Meis2* by ChIP qPCR. Enrichment of each region following immunoprecipitation with Hoxa2 and IgG negative control antibody (Neg Ab) is calculated as percentage input; 1 and 2 indicate the corresponding peaks in (A). *Pou6f2* is a positive control and *Itih4* is a negative control (unbound region). Values represent the average of duplicate samples, and error bars indicate the SEM.

(C–F) Whole-mount ISH on E11.5 wild-type (C and E) and *Hoxa2* mutant (D and F) embryos, using *Meis1* and *Meis2* probes. Arrows indicate the proximal domain of expression in the IIBA. See also Figure S1.

To understand how Hox operate in vivo, we mapped Meis, Pbx, and Hoxa2 binding in the native area of competence of Hoxa2, the IIBA. Through this analysis we uncovered a synergistic interaction between Hoxa2 and Meis TFs. Collectively, our findings show that Meis TFs provide a ground state that is common to all segments (arches). Hoxa2 recognizes a subset of Meis prelabeled sites, which contain Hox recognition motifs. By selectively binding to and enhancing a subset of Meis binding, Hoxa2 modifies the branchial arch ground state, established by Meis, to instruct IIBA-specific morphologies. This specific synergy between Hoxa2 and Meis is further reinforced by a positive-feedback loop, which locks IIBA cells in a state of high *Hoxa2* and *Meis* levels. Our results highlight the importance of genomic profiling TFs in their native, tissue-specific expression domains to understand the mechanisms governing segment-specific identity.

RESULTS

Hoxa2 Activates *Meis* Genes

Hoxa2 controls the correct development of the IIBA (Gendron-Maguire et al., 1993; Rijli et al., 1993), most likely by binding to and regulating the expression of target genes. By analyzing Hoxa2 chromatin immunoprecipitation sequencing (ChIP-seq) (Donaldson et al., 2012), we observed frequent binding of Hoxa2 to the genomic regions that contain *Meis1* and *Meis2* (13 and 15 Hoxa2-bound regions were associated with *Meis1* and *Meis2*, respectively; the average Hoxa2-bound regions per gene = 2.7). We extracted chromatin from the IIBA of E11.5 embryos and confirmed that Hoxa2 binds to *Meis1* and *Meis2* (Figures 1A and 1B). At the same embryonic stage (E11.5), whole-mount in situ hybridization (ISH) revealed high expression of *Meis1* and *Meis2* in the main *Hoxa2* domain of expression, the IIBA, and in the posterior margin of the IBA (Figure S1 available online). We found that both transcripts were downregulated, and the proximal domains of *Meis1* and *Meis2* expression were absent in the IIBA of *Hoxa2* mutant embryos (Figures 1C–1F, arrows). The binding of Hoxa2 to *Meis1* and *Meis2* and their downregulation in *Hoxa2* loss-of-function embryos strongly suggest that Hoxa2 is directly upstream of *Meis1* and *Meis2* in vivo.

Meis Transcription Factors Control the Formation of the Visceral Skeleton

The above results suggest that Meis1/2 TFs are part of the gene regulatory network controlled by Hoxa2 to instruct the IIBA fate. *Meis1* mutant mice display neither abnormalities in CNC derivatives nor Hox-related phenotypes (Azcoitia et al., 2005; Hisa et al., 2004), possibly due to the redundant functions of *Meis2* (*Meis3* is not expressed in the branchial arches). Therefore, because Meis1 and 2 are highly conserved in vertebrates (Longobardi et al., 2014), we used a zebrafish embryo model to systematically deplete Meis proteins. After injecting one-to-two-cell-stage embryos with morpholino oligonucleotides targeting translation of Meis transcripts expressed in the IIBA (*meis1*, *meis2a*, *meis3*, and *meis4.1a*) (Thisse and Thisse, 2005), we observed an almost complete absence of the visceral, neural crest-derived skeleton, including the skeleton derived from the *Hoxa2*-positive domain (Figures 2A and 2B; 14/14 embryos affected). Abnormalities in the skeletal derivatives of the branchial arches also were observed in embryos injected with a dominant-negative construct, which interfered with Meis nuclear entry (data not shown). Our findings are consistent with

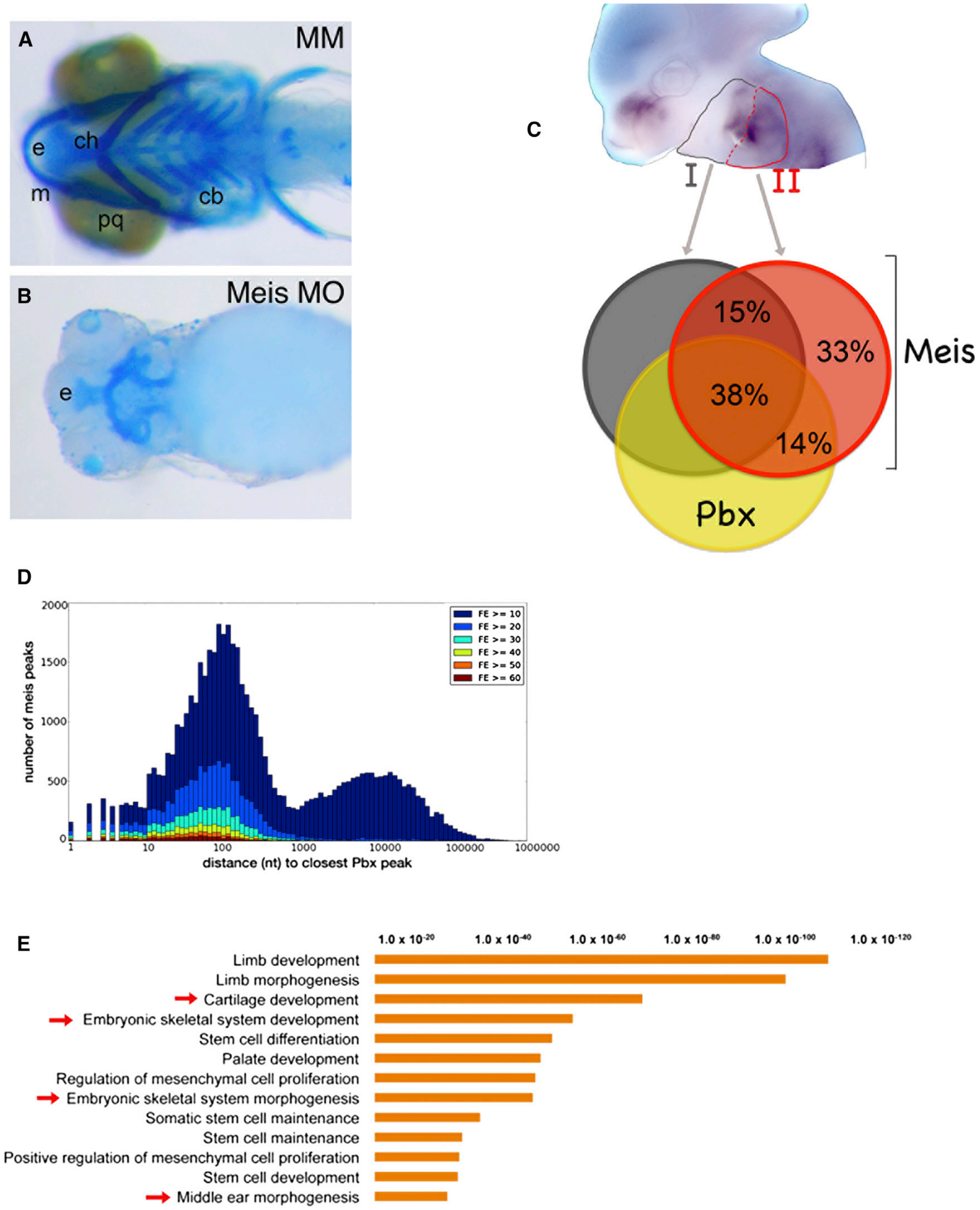

Figure 2. Meis TFs Are Required to Form the Branchial Arch-Derived Skeleton

(A and B) Ventral view of zebrafish larval (6 days postfertilization) control (mismatched morpholino, MM) (A) and Meis-morpholino-injected embryos (B) head skeleton. The IIBA-derived skeleton (ceratohyal) is labeled by ch.

(C) Craniofacial region of a E11.5 mouse embryo hybridized with *Meis1* antisense probe; IBA (gray) and IIBA (red) are outlined. Overlap of Meis summit regions (200 nt, overlap at least 1 nt) in the IIBA (red), with Meis summit regions in the IBA (dark gray) and Pbx summit regions in the IIBA (yellow).

(D) Distance of Meis peaks relative to Pbx peaks. Meis peaks (IIBA) are binned according to the distance to the nearest Pbx peak and labeled according to FE (high FE, dark red bars; low FE, dark blue bars).

(E) Top overrepresented functional categories associated to common Meis/Pbx-bound regions in the branchial arches. The length of the bars corresponds to the binomial raw (uncorrected) p values (x axis values). Cb, ceratobranchials; ch, ceratohyals; e, ethmoid plate; m, Meckel cartilage; and pq, palatoquadrate. See also Figure S2.

the identification of *Meis1* and *Meis2* as primarily involved in the formation of the viscerocranium in zebrafish (Melvin et al., 2013), and mirror the inactivation of Prep1.1, another TALE family member (Deflorian et al., 2004). Collectively, these observations indicate that *Meis* genes are essential for the development of the entire branchial arch-derived skeleton in zebrafish.

To identify the molecular mechanisms controlled by Meis1/2 in the branchial arches, we mapped Meis genomic occupancy in both Hox-negative (IBA) and Hox-positive (IIBA) arches in mouse. Meis bound many regions (>60,000 regions) in both Hox-positive (II) and -negative (I) branchial arches (Tables S1 and S2), which were widely distributed across the genome (Figures S2A and S2B). Importantly, we found that a large fraction of Meis binding (>30,000 Meis-bound regions) were common to both Hox-positive (II) and -negative (I) branchial arches, and largely overlapped Pbx binding in the IIBA (Figure 2C; Table S3). These binding overlaps are close in size to the binding overlaps expected across biological replicates of genome-wide-binding experiments (>50%) (Bardet et al., 2012). Indeed, the majority of Meis binding, including the highest Meis peaks, occurred within 1 kb of Pbx binding; unrelated binding to Pbx mainly consisted of low-enrichment binding (Figure 2D). According to gene ontology terms analysis, Meis and Pbx combinatorial binding in the branchial arches mapped close to genes involved in skeletal development (Figure 2E, red arrows), stem cell maintenance and differentiation, and the IBA- and IIBA-specific process middle ear morphogenesis (Figure 2E, red arrows). In sum, Meis TFs occupy a large pool of common regions in the branchial arches and control the formation of the entire visceral skeleton, which derives from both the Hox-negative and Hox-positive branchial arches. Collectively, these observations suggest that Meis TFs have a widespread regulatory role in the CNC.

Meis Regulates *Hoxa2* in the IIBA

The differential expression of Hox genes along the anteroposterior axis of the embryo is imposed by their relative positions in the chromosome and is crucial for Hox patterning activities (Duboule and Dollé, 1989; Graham et al., 1989). In the branchial arches, members of the paralog group 2 (*Hoxa2 and Hoxb2*) are expressed in arch II, and paralogs of group 3 are expressed in arch III, while the first arch does not express any Hox genes (Hunt et al., 1991). We observed strong Meis binding at the HoxA cluster in IIBA-chromatin, peaking at the *Hoxa2* promoter and stretching to the neighboring *Hoxa1*/*Hoxa3* genes (Figure 3A). In the HoxB cluster, Meis binding was restricted to the *Hoxa2* paralog *Hoxb2* (albeit with a lower peak) (Figure 3A). In contrast, we did not detect binding of Meis to the HoxA or HoxB clusters in the adjacent anterior IBA, where Hox genes are not expressed (Figure 3A). In sum, Meis binding appears to specifically mark actively transcribed areas of the Hox clusters. Confirming these results, we found that the *Hoxa2* proximal promoter was specifically enriched in IIBA chromatin after immunoprecipitation with Meis antibodies, but not when chromatin was extracted from the adjacent, Hox-negative IBA (Figure 3B). The Meis binding located upstream of the *Hoxa2* gene (including the highest Meis peak overlapping the *Hoxa2* proximal promoter) was contained in a 4.0 kb fragment sufficient to drive gene expression in the hindbrain rhombomere 3 (r3) and r5 and in the CNC migrating from r4 into the IIBA (Nonchev et al., 1996). In a cotransfection assay, *Hoxa2* proximal promoter showed the strongest transactivation when Hoxa2 and Meis1 were used together (Figure 3C). The *Hoxa2* proximal promoter was extremely conserved across vertebrates (Figure 3D), and, as in the mouse, the zebrafish *hoxa2b* proximal promoter was highly enriched in chromatin immunoprecipitated with Meis3 antibodies (Figure 3E).

Having established that Meis binding to the *Hoxa2* proximal promoter is conserved from mouse to zebrafish, we turned to the zebrafish embryo model to examine the effects of Meis knockdown on *Hoxa2* expression. After injecting one-to-two-cell-stage embryos with morpholino oligonucleotides targeting translation of meis transcripts expressed in the branchial arches, we observed a downregulation of *hoxa2b* expression in the hindbrain and IIBA of all embryos (39/39 affected). These embryos still expressed *dlx2*, which labeled the developing branchial arches (Figure 3F). In sum, our results indicate that Meis TFs control *Hoxa2* expression and that this mechanism is conserved in vertebrates. Together with the converse activation of *Meis1/2* by Hoxa2, this mechanism defines a positive-feedback loop that maintains and amplifies *Hoxa2* expression in the IIBA. It also secures high levels of *Hoxa2* and *Meis* transcripts in the same cells of the IIBA.

Hoxa2 Largely Binds Close to TALE Proteins

The prevailing view is that TALE homeodomain proteins act as ancillary cofactors for Hox; however, these interactions have yet to be mapped in vivo on a genome-wide scale. For this reason, and to fully understand the role of Meis TFs in *Hoxa2*-positive domain, we investigated whether Hoxa2 occupied common *cis*-regulatory modules with Pbx and Meis in the IIBA.

We found that the majority of Hoxa2 binding clustered within 1 kb of Meis binding. The highest Hoxa2 peaks occurred within a closer (200 nt) distance from Meis binding, and only a small fraction of Hoxa2-binding events was apparently unrelated to Meis binding (Figure 4A). A similar distribution was observed with Pbx (Figure S3A). Hoxa2 combinatorial binding largely involved the three factors (Figure 4B). Chromatin accessibility is a major determinant of TFs binding (Biggin, 2011). By mapping the binding of Foxc1 (an unrelated, nonhomeodomain TF) in E11.5 IIBAs, we observed that the binding overlap of Hoxa2 and Foxc1 was significantly lower than that of Hoxa2 and Meis (Figures S3B–S3D). Similarly, Meis bound at a significantly higher frequency with Pbx than with Foxc1 (Figure S3E), indicating that the extensive binding overlap of Hox and TALE proteins is determined by other factors in addition to the chromatin structure.

Functional annotation of Hoxa2/Meis/Pbx-shared regions identified enrichment of genes in overlapping functional categories with the entire Hoxa2 ChIP-seq (Figure 4C), while the genomic regions enrichment of annotations tool (GREAT) analysis of Hoxa2 unique binding sites did not detect association with any functional processes (not shown). Regions occupied by Hoxa2, Meis, and Pbx displayed a higher sequence conservation compared to the entire Hoxa2 ChIP-seq (Figure 4D). Collectively, these observations indicate that Hoxa2 largely binds in combination with Meis and Pbx in the IIBA, and suggest that combinatorial binding underlies Hoxa2 function.

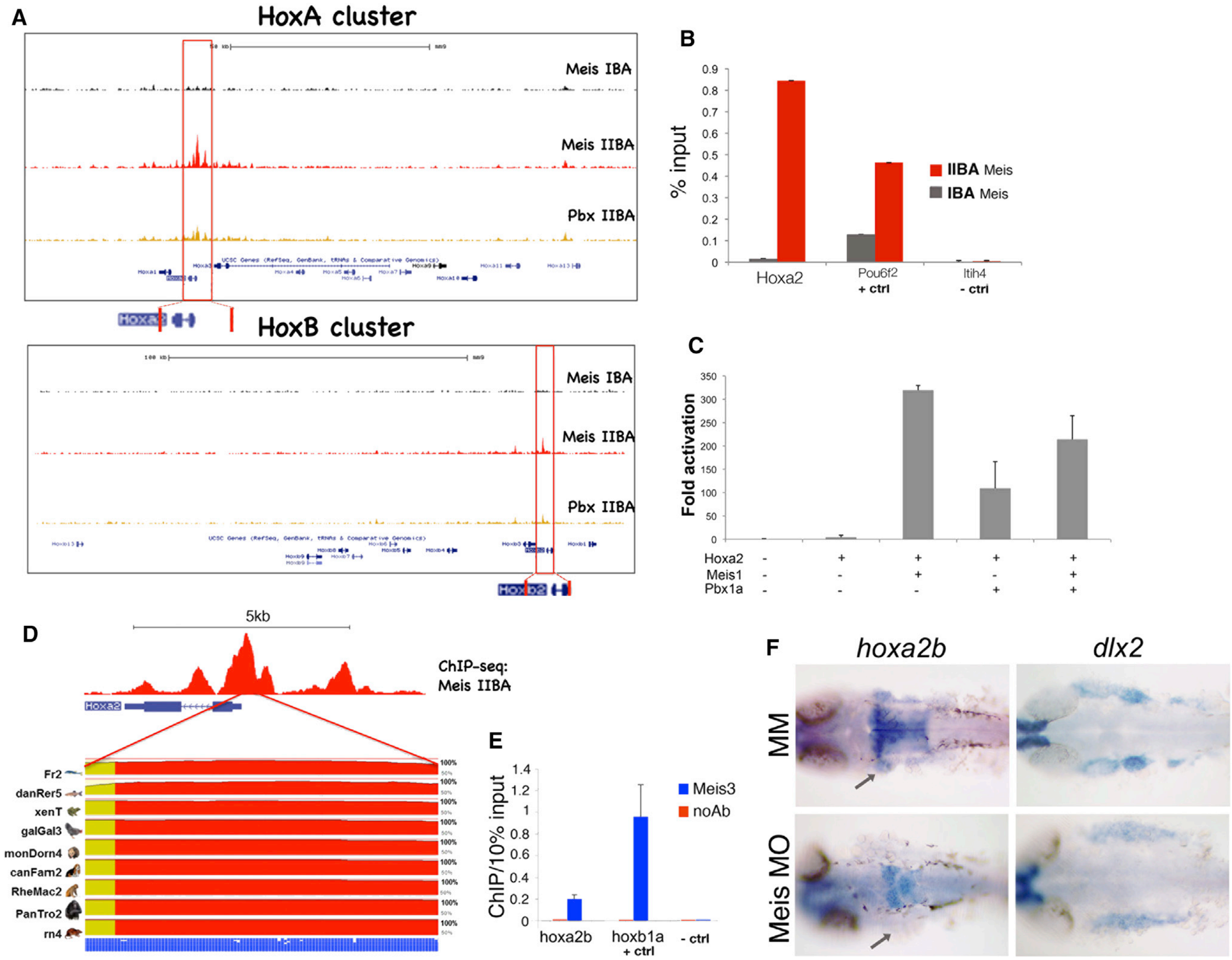

Figure 3. Meis Regulates *Hoxa2* Expression

(A) Meis and Pbx ChIP-seq-binding profiles at the HoxA and HoxB clusters in the IBA and IIBA of E11.5 embryos. Red boxes highlight Meis binding at *Hoxa2* and *Hoxb2* in IIBA chromatin.

(B) Meis occupancy at *Hoxa2* promoter in IBA and IIBA chromatin (mouse) by ChIP qPCR. *Pou6f2* is a positive control and *Itih4* is a negative control (unbound region). Values represent the average of duplicate samples, and error bars indicate the SEM.

(C) Luciferase activity driven by *Hoxa2* proximal promoter in HEK293T cells alone or in combination with Hoxa2, Meis, and Pbx expression vectors. Values represent fold activation over basal promoter activity, and are presented as the average of at least two independent experiments, each performed in triplicate. Error bars represent the SEM.

(D) Sequence conservation of the *Hoxa2* proximal promoter in vertebrates, generated by the ECR Browser (Ovcharenko et al., 2004).

(E) Meis binding at *hoxa2b* promoter in zebrafish embryos by ChIP qPCR. *Hoxb1a* is a positive control; the negative control is a genomic region 10 kb upstream of the *hoxba* cluster. Enrichment of *hoxa2b* and *hoxb1a* is significantly higher compared to the negative control regions ($p < 0.005$). Values represent the average of three independent experiments, and error bars indicate the SEM.

(F) Whole-mount ISH on control MM and Meis-morpholino-injected embryos, using *hoxa2b* and *dlx2* probes. *Hoxa2b* is downregulated in the second arch (gray arrows); *dlx2* labels the developing branchial arches.

Hox proteins form dimers with either a Pbx protein or a Meis protein, as well as trimers with one Pbx protein and one Meis protein (reviewed in Mann et al., 2009). Because complex formation improves the affinity and sequence selectivity of Hox proteins, we asked if Hoxa2/TALE complexes occupy different genomic regions relative to TALE proteins alone. Noticeably, the large majority (67%) of the regions occupied by Hoxa2 with Meis and Pbx in the IIBA overlapped with regions bound by Meis in the IBA, where Hoxa2 is absent (Figure 4E), suggesting that Hoxa2 binds Meis-prelabeled sites. Thus, binding of Meis, possibly with Pbx, provides an accessible chromatin platform for Hoxa2 to bind.

Hoxa2 Enhances Meis Binding in the IIBA

Next, we investigated whether the presence of Hoxa2 affects Meis binding to chromatin. As Hoxa2 does not alter the spatial occupancy of Meis TFs in the IIBA, we focused on the binding signal of Meis peaks in Hoxa2-positive (IIBA) and

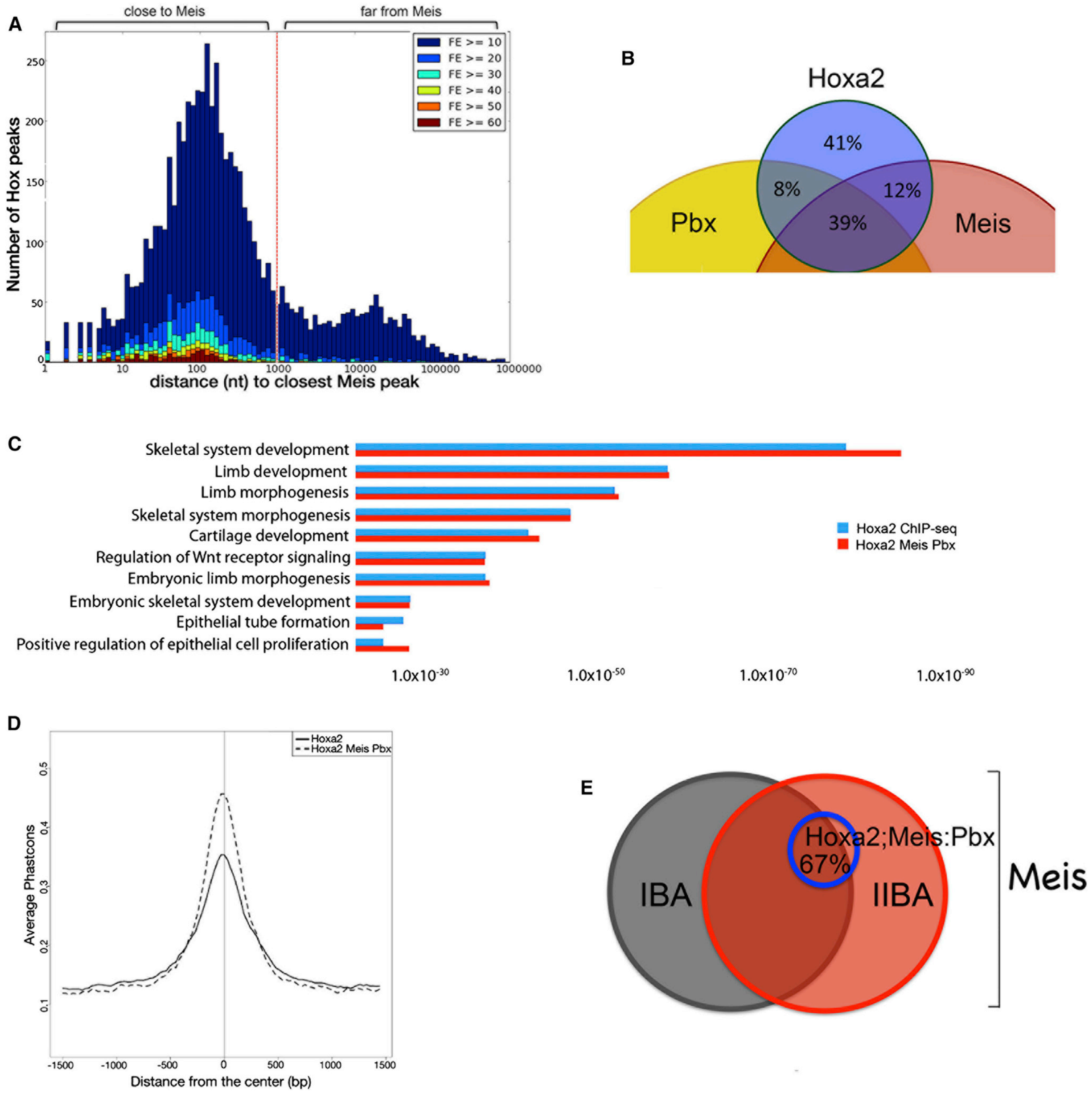

Figure 4. Combinatorial Binding of Hoxa2, Meis, and Pbx

(A) Distance of Hoxa2 peaks relative to Meis peaks. Hoxa2 peaks are binned according to distance to the nearest Meis peak and labeled according to FE (high FE, dark red bars; low FE, dark blue bars).

(B) Binding overlap of Hoxa2 with Meis and Pbx in the IIBA (200 nt summit regions, overlap at least 1 nt). Only the overlapping portion of the larger Pbx and Meis data sets has been included in the figure.

(C) Functional categories identified by GREAT analysis of whole Hoxa2 ChIP-seq data set (blue bars) and Hoxa2/Meis/Pbx-shared regions (red bars). The length of the bars corresponds to the binomial raw (uncorrected) p values (x axis values).

(D) Average sequence conservation (vertebrates) of Hoxa2 binding (entire Hoxa2 ChIP-seq, continuous line) and Hoxa2 combinatorial binding with Meis and Pbx (dashed line), centered on the summit of the peaks.

(E) Overlap of Hoxa2/Meis/Pbx combinatorial binding in the IIBA (blue circle) with Meis binding in the IBA (dark gray) and Meis binding in the IIBA (red). See also Figure S3.

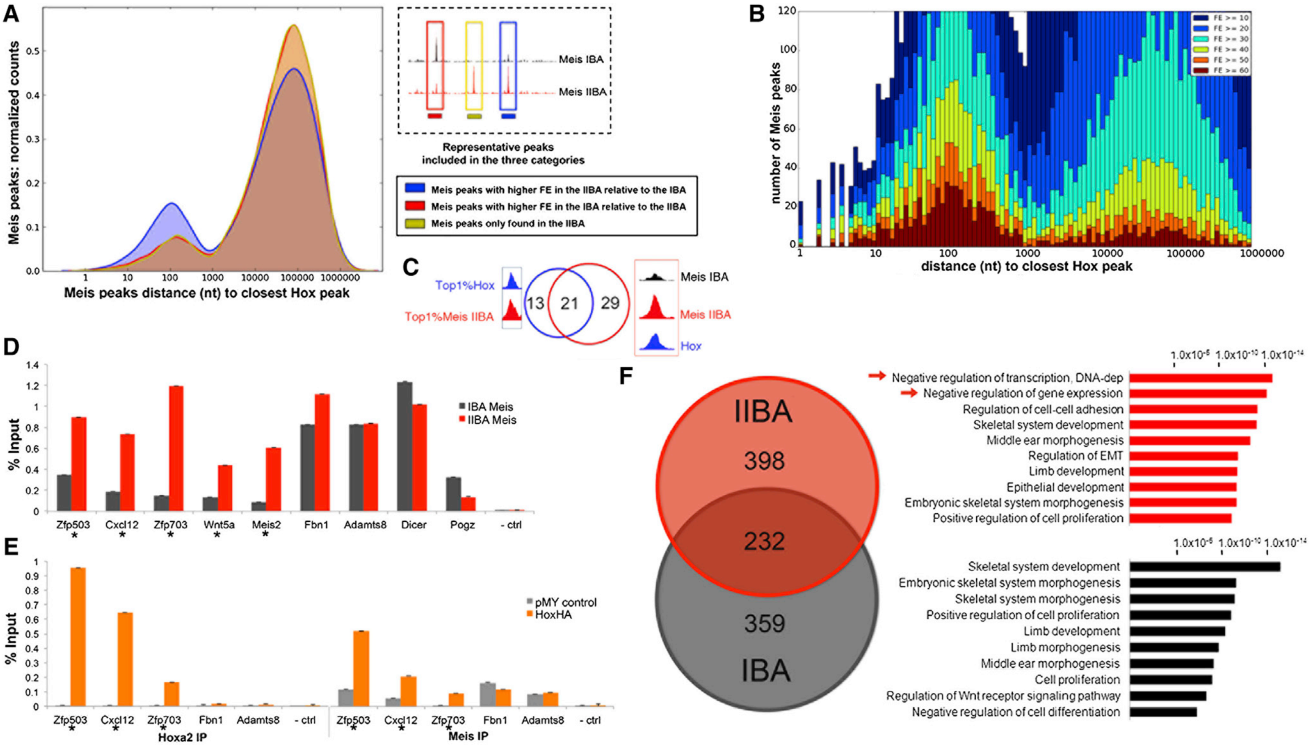

Figure 5. Hoxa2 Enhances Meis Binding

(A) Overlay density plots of Meis binding relative to the distance of the nearest Hoxa2 peak. Meis binding was sorted into (1) peaks with higher FE in the IIBA relative to the IBA (blue), (2) Meis peaks with higher FE in the IBA relative to the IIBA (red), and (3) Meis peaks only found in the IIBA (yellow).

(B) Distance of Meis peaks (IIBA) relative to Hoxa2 peaks. Meis peaks are binned according to their distance to the nearest Hoxa2 peak and labeled according to FE. The histogram is cropped to focus on high Meis peaks; a full version is shown in Figure S4.

(C) Strategy used to identify high-confidence Meis/Hoxa2 synergistic binding. Intersection of top Meis peaks overlapping top Hoxa2 peaks (200 nt summit regions, exemplified in the blue rectangle; n = 34) with Meis peaks with higher binding signal in the IIBA relative to the IBA and overlapping a Hoxa2 peak (200 nt summit regions, exemplified in the red rectangle; n = 50). Both sets of sequences are likely to be enriched in synergistic binding events; their intersection (Venn diagram) resulted in 21 regions, referred to as Meis/Hoxa2 synergistic binding regions (listed in Table S4).

(D) Meis occupancy in the IBA (gray bars) and IIBA (red bars) by ChIP qPCR. Meis/Hoxa2 synergistic binding regions are indicated by asterisks; *Fbn1*, *Adamts8*, *Dicer*, *and Pogz* are control regions (where Meis binding does not overlap Hoxa2 binding); and *Ith4* is a negative control region. Percentage input is shown for IBA and IIBA. Values represent the average of duplicate samples and error bars represent the SEM.

(E) Binding of Meis and Hoxa2 in IBA cells infected with pMY control (gray) or pMY-Hoxa2-HA (orange) by ChIP qPCR. Asterisks indicate Hoxa2/Meis synergistic binding regions; *Fbn1* and *Adamts8* are control regions; and *Ith4* is a negative control region. Values represent the average of duplicate samples and error bars indicate the SEM.

(F) Overlap of top 1% Meis peaks in the IBA with top 1% Meis peaks in the IIBA and corresponding overrepresented functional categories identified by GREAT analysis. Shared functional categories in IBA and IIBA contain regions bound by Meis in both tissues (232 common regions) and regions bound by Meis in one tissue (IBA or IIBA) and associated to genes sharing the same GO. The length of the bars corresponds to the binomial raw (uncorrected) p values (x axis values). See also Figure S4.

Hoxa2-negative (IBA) branchial arches. Meis peaks with a higher binding signal in the IIBA (measured by fold enrichment [FE]) were preferentially located close (200 nt) to Hoxa2 binding (Figure 5A), suggesting that the presence of Hoxa2 enhances Meis occupancy on chromatin. Next, we examined the entire distribution of Meis binding in the IIBA relative to Hoxa2 binding. Whereas the vast majority of Meis binding was apparently unrelated to Hoxa2 (Figure S4A), we observed that high Meis peaks (FE > 40) preferentially occurred close to Hoxa2 binding, with a marked tendency for top Meis peaks (FE > 60) to occur close to a Hoxa2-binding event (Figure 5B). In contrast, we found that high Pbx and high Foxc1 peaks were equally distributed close to and far from Hoxa2 (Figures S4B–S4D), and occurred at a significantly lower frequency close to a Hoxa2-binding event than high Meis peaks (Figure S4E). In line with these observations, we found that Meis binding at a subset of regions bound by Hoxa2 in the IIBA (Donaldson et al., 2012) was markedly increased in the IIBA compared to the IBA, while Pbx binding was only modestly affected (Figure S4F). In summary, these observations suggest that Hoxa2 enhances Meis binding to chromatin. Reciprocally, the highest Hoxa2 peaks occurred within 200 nt of Meis binding (Figure 4A), suggesting that Hoxa2 and Meis proximity reinforces their binding to chromatin. We name this effect, which may result from cooperativity, Hoxa2 and Meis synergistic binding.

We selected a set of high-confidence, synergistic binding events to test if Hoxa2 affects binding of Meis (Figure 5C). First, we confirmed that Meis binding is increased at these regions in

the IIBA (Figure 5D). Upon expressing Hoxa2 in Hox-negative IBA cells (Anderson et al., 2013; Kirilenko et al., 2011), we observed increased levels of Meis binding, and high levels of Hoxa2 binding, at these high-confidence regions. In contrast, Meis binding levels remained unaffected at regions that were not bound by Hoxa2 (Figure 5E). In this system, the Hoxa2/Meis positive-feedback loop was inactive: Meis did not bind to the Hox clusters (Figure 3A), and Hox genes were not transcribed (Hoxa2 was ectopically expressed using a heterologous promoter). As a result, Meis transcript levels were only modestly increased by Hoxa2 in this system (no change in *Meis1*; 1.3-fold change in *Meis2*) (Anderson et al., 2013) and were unlikely to account for the increase in Meis-binding levels. We therefore conclude that Hoxa2 binding specifically enhances binding of Meis.

Finally, we asked whether enhancement of Meis binding in the IIBA reflects biologically meaningful differences in the branchial arches. We focused on the top 1% of Meis peaks for analysis (containing peaks with FE > 60), as only high-FE Meis-bound regions showed a skewed distribution relative to Hoxa2 distance (Figure 5B). Functional annotation of arch-specific high-confidence Meis binding showed an association with different biological processes in the IBA and IIBA (Figure 5F). Top Meis-binding events in the IIBA (n = 630) mapped close to genes that negatively regulate transcription/gene expression. These terms were exclusively enriched within the fraction of Meis peaks that overlap Hoxa2 binding (n = 347), and not with top Meis peaks unrelated to Hoxa2 in the IIBA (n = 283) (Figure S4G) or top Meis peaks in the IBA (cutoff $p < 1 \times 10^{-3}$). GREAT showed that top Meis binding in both branchial arches was associated with genes involved in skeletal development, consistent with the control of skeletal development by Meis TFs. Association with middle ear morphogenesis was also detected, which is consistent with the middle ear forming from both IBA and IIBA. We observed the same differential association using the top 1% of Meis ChIP-seq replicates in the IBA and IIBA (data not shown). In sum, the enhanced Meis binding in the IIBA, caused by Hoxa2, appears to target specific biological processes in the IIBA.

Synergistic Binding of Hoxa2 and Meis Is Sequence Specific

We interrogated DNA sequence motifs to identify the mechanisms underlying synergistic binding of Hoxa2 and Meis in the IIBA. We found TGACAG, which corresponds to the canonical Meis recognition motif, as the most highly overrepresented motif in the entire Meis ChIP-seq and in highly enriched Meis peaks unrelated to Hoxa2 binding (FE > 70, closest Hoxa2-binding event > 10 kb; n = 78) (Figure 6A). In contrast, scanning Meis summit regions corresponding to synergistic binding (Figure 5C; Table S4) identified the reverse complement of GATNNAT (Figure 6A), an almost perfect match of the Hox-Pbx recognition motif (TGATNNAT). In agreement with de novo motif discovery, Meis summit regions corresponding to synergistic binding with Hoxa2 displayed a significantly higher occurrence of GATNNAT (76%; 16/21) (Figure 6B), and also a high occurrence of the single Hox motif TAAT (present in 19/21 sequences with an average 3 TAAT/peak). The occurrence of the Meis recognition motif TGACAG was similar to the entire Meis ChIP-seq, but most Meis/Hoxa2 synergistic binding regions contained a partial Meis consensus, TGACA (86%; 18/21). Collectively, these observations suggest that the presence of closely arranged recognition motifs is important to bring Hoxa2 and Meis together. Indeed, we observed a high occurrence of GATNNAT in the entire set of Meis summits overlapping Hoxa2 binding (48%; 2,457/5,115). Further supporting this conclusion, TGACAG was identified as the most recurrent flanking motif to TGATNNAT in Hoxa2 ChIP-seq (Donaldson et al., 2012). Moreover, Hoxa2 summit regions (200 nt) enriched in Meis and Hox recognition motifs (≥1 GATNNAT; ≥1 TGACAD; ≥3 TAAT; average sequence conservation ≥ 40%; n = 450) showed association with the gene ontology (GO) terms negative regulation of transcription and negative regulation of gene expression (p value = 1×10^{-15}), which also were enriched in the fraction of top Meis binding in the IIBA. In sum, these observations indicate that the assembly of Hox-Meis complexes is sequence based.

Cooperation of Meis and Hoxa2 at Hoxa2-Regulated Genes

Finally, we examined whether the selective enhancement of Meis binding in the IIBA underlies Hoxa2 function. As a direct readout of Hoxa2 activity, we used the changes in gene expression detected in the IIBA in the absence of Hoxa2 (Donaldson et al., 2012). We observed a strong positive correlation between Hoxa2/Meis synergistic binding and genes activated by Hoxa2. Only a small fraction of Hoxa2-binding events (8% of Hoxa2 ChIP-seq) was associated with genes regulated by Hoxa2, while up to 48% of Meis/Hoxa2 synergistic binding (Figure 5C; Table S4) was associated with genes dysregulated in *Hoxa2* mutants (Figure 6C; Figure S5A). In all cases, genes displayed a decrease in expression in the *Hoxa2* mutant, suggesting that Meis/Hoxa2 synergistic binding defines enhancer regions. The majority of Meis/Hoxa2 synergistic binding was located far away (>50 kb) from genes (Figure S5B), and all the regions tested displayed high enrichment of the histone mark H3K27Ac, which maps active enhancers (Figure 6D). Further suggesting that Meis/Hoxa2 synergistic binding labels IIBA long-range enhancers, insertions of a reporter gene in topologically associated domains (Chen et al., 2013; Dixon et al., 2012), which contain Hoxa2/Meis synergistic binding, highlighted the presence of regulatory domains of IIBA-specific expression (Figure 6E; Figure S5C). These domains of expression correspond to the expression of distant but associated Hoxa2-regulated genes (Figure 6E). These observations strongly suggest that Hoxa2 and Meis bind cooperatively to activate transcription. Supporting this conclusion, Meis has a positive effect on Hox-dependent transcription (Choe et al., 2009).

We therefore focused on the genes linked to synergistic binding of Hoxa2 and Meis (Table S4). *Meis2* and *Wnt5a* were highly expressed in the IIBA and downregulated in *Hoxa2* mutant embryos (Figures 1C–1F; Donaldson et al., 2012). *Wnt5a* is required for the formation of the pinna, a IIBA-specific derivative (Donaldson et al., 2012; Minoux et al., 2013; Yamaguchi et al., 1999). Interestingly, a few of these genes were included in the GO category negative regulation of transcription/gene expression, which is specifically associated with high Meis peaks in the IIBA (*Zfp703*, *Zfp503*, and *Wnt5a*). *Zfp703* and *Zfp503* encode for two related zinc-finger proteins that act as transcriptional repressors. In zebrafish, they are required for the formation of r4

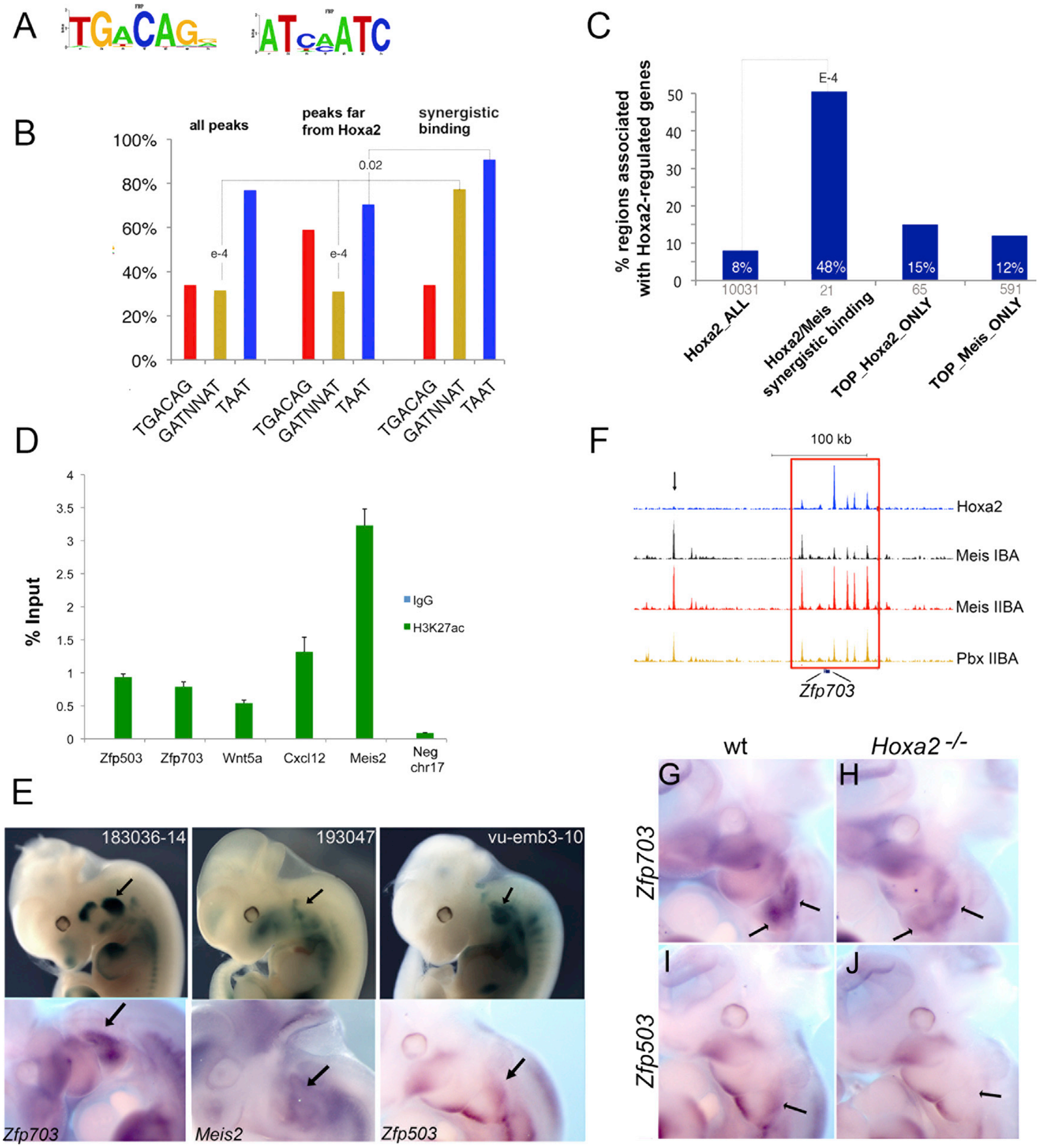

Figure 6. Synergistic Binding Is Sequence Based and Associated with Hoxa2-Activated Genes

(A) Sequence logo of the top motifs identified using de novo motif discovery.

(B) Distribution of motifs in Meis peaks (entire ChIP-seq), top Meis peaks far from Hoxa2, and Meis peaks corresponding to Hoxa2-Meis synergistic binding (200 nt summit regions). Red, yellow, and blue columns represent the occurrence of TGACAG, GATNNAT, and TAAT, respectively. The occurrence of GATNNAT is significantly higher in synergistic binding events relative to Meis ChIP-seq (p values shown on the tops of columns).

(C) Percentage of Hoxa2-bound regions associated to a Hoxa2-regulated gene in the entire Hoxa2 ChIP-seq (Hoxa2_ALL), Hoxa2/Meis synergistic binding, top 1% Hoxa2 peaks (TOP_Hoxa2_ONLY), and top 1% Meis peaks (TOP_Meis_ONLY). For each category, the corresponding number of regions is indicated on the x axis.

(D) High enrichment of the histone mark H3K27Ac on Hoxa2/Meis synergistic binding regions in IIBA chromatin, relative to a negative control region using ChIP qPCR. Data are presented as the average of two independent experiments in duplicate and error bars indicate the SEM.

(E) Integration of a *lacZ* reporter gene in genomic regions containing Hoxa2/Meis synergistic binding events. The expression of the reporter (top) and the expression of Hoxa2-regulated genes associated to the integration sites (bottom) are shown.

(F) ChIP-seq tracks corresponding to the genomic region containing *Zfp703*. Meis binding in the IIBA overlapping a Hoxa2 peak (enclosed by the red rectangle) is enhanced relative to Meis binding in the IBA. Black arrow shows similar binding of Meis in the IBA and IIBA in regions not bound by Hoxa2.

(G–J) Whole-mount ISH on E11.5 wild-type (G and I) and *Hoxa2* mutant (H and J) embryos, using *Zfp703* (G and H) and *Zfp503* (I and J) probes. Both *Zfp703* and *Zfp503* are specifically downregulated in the IIBA (black arrows) of *Hoxa2* mutant embryos. See also Figure S5.

and the expression of r4-specific genes, including *hoxa2* (Nakamura et al., 2004, 2008; Runko and Sagerström, 2003, 2004).

A survey of *Zfp703* and *Zfp503* genomic regions revealed a high density of Hoxa2- and Meis-binding events, and a higher Meis-binding signal in the IIBA in locations that were cooccupied with Hoxa2 (Figure 6F). We examined the expression of *Zfp703* and *Zfp503* and found that both genes were highly expressed in the IIBA (Figures 6G and 6I, arrows) and specifically downregulated in the IIBA of *Hoxa2* mutant embryos (Figures 6G–6J). In addition, consistent with their transcription being directly regulated by Hoxa2, both genes were upregulated when *Hoxa2* was overexpressed in the IBA (Anderson et al., 2013). In sum, synergistic binding of Meis and Hoxa2 results in enhancer activity associated with genes that are strongly expressed in the IIBA and regulated by Hoxa2. Taking into account that Meis is essential to form the skeleton of the branchial arches, and that the presence of Hoxa2 selectively reinforces Meis binding to regions linked to Hoxa2-activated genes, we hypothesize that, by enhancing Meis binding, Hoxa2 modifies a basal skeletal program controlled by Meis in the branchial arches to construct the second arch-specific skeleton.

DISCUSSION

It is clear that Hox genes specify the identities of embryonic segments, yet how they do it is still poorly understood. Our results show that Hoxa2 selectively enhances a ground-state binding of Meis in the branchial arches to instruct the IIBA-specific identity. Reinforcing Meis binding at selected enhancers can generate large phenotypic differences, because these enhancers regulate transcriptional repressors.

These findings change our interpretation of Hox/TALE cooperative binding. Rather than simply increasing DNA-binding specificity, binding with TALE enables Hox to modify the function of TALE and change a branchial arch ground state established by TALE to generate specific anatomical identities.

TALE Proteins Recruit Hoxa2 to Chromatin

Our results provide a molecular explanation for the recruitment of Hoxa2 to chromatin. Hoxa2 occupancy largely overlaps with Meis binding in the branchial arches, indicating that Meis creates an accessible platform that is recognized by Hoxa2. While chromatin accessibility is a main determinant of TF binding (Biggin, 2011), chromatin structure and indirect cooperativity (increase in the occupancy of a TF caused by other proteins' binding and partial displacement of the nucleosome from the DNA) (Polach and Widom, 1996) do not entirely explain the extensive overlap of Hoxa2 and TALE.

Because Hoxa2 can interact with Meis (Williams et al., 2005), it is highly likely that Hoxa2 is recruited to chromatin by direct interaction with prebound Meis, and possibly Pbx. Collectively, the observations that Pbx acts as a pioneer factor and that it largely binds with Meis in the branchial arches and in the entire embryo (Penkov et al., 2013) suggest that Pbx could be the first determinant for Meis binding. The similar distribution of Hoxa2 peaks relative to Meis and to Pbx binding (Figure 4A; Figure S3) likely reflects the recruitment of Hoxa2 by both Meis and Pbx, or alternatively a requirement of prebound Pbx for Meis binding. Dissecting the sequence of the binding events responsible for loading Meis/Pbx complexes onto chromatin, and the precise temporal order of such events, will require additional experiments. Supporting an active role for Pbx, *Pbx1*-null mutant embryos display IIBA defects (Selleri et al., 2001); additionally, Pbx is required for Hox and Meis binding to DNA in vitro (Longobardi et al., 2014) and is ubiquitously expressed (Yokoyama et al., 2009). Within the accessible platform provided by TALE proteins, Hoxa2 selects a subset of Meis/Pbx-prelabeled regions that contain a Hox recognition motif.

Hoxa2 and Meis Cooperative Binding

While Meis and Pbx similarly promote Hoxa2 binding, Hoxa2 exerts a reciprocal effect on Meis, but not Pbx. Enhanced Meis binding is observed close to Hoxa2 in the IIBA, at sites highly enriched in Meis and Hox recognition motifs. Importantly, the addition of Hoxa2 to Hox-negative cells reinforces Meis binding at selected sites. Collectively, these observations suggest that Hoxa2 and Meis bound to adjacent sites on DNA could reinforce each other's occupancy by direct protein-protein interactions (Spitz and Furlong, 2012; Williams et al., 2005), although indirect mechanisms (e.g., the requirement of additional proteins that bridge Hoxa2 and Meis) cannot be excluded at this stage.

A range of possibilities likely contributes to higher Meis binding levels. Because TFs exchange rapidly between the DNA-bound and -unbound states, higher Meis binding levels could reflect a longer residence time at the same genomic location in the presence of Hoxa2 (e.g., Meis-binding events are stabilized by interactions with Hoxa2). In addition, the synergy between Hoxa2 and Meis is reinforced by genetic interaction. A positive-feedback loop expands *Meis* expression domain to Hoxa2-positive cells in the IIBA, resulting in more cells displaying Meis binding at the same sites in the IIBA relative to the IBA. In addition, the resulting higher levels of Meis1/2 and Hoxa2 in IIBA cells could allow formation of a stable Hoxa2/Meis complex on sites that may not have optimal Meis-binding sites per se.

Reciprocal Meis/Hox activation appears to be a broadly used, possibly general mechanism: *XMeis3* activates Hox genes in *Xenopus* (In der Rieden et al., 2011) and anterior Hox and *Meis2* are concomitantly induced by retinoic acid (Colberg-Poley et al., 1985; Oulad-Abdelghani et al., 1997).

Hoxa2 Specifies IIBA Identity by Reinforcing a Ground-State Binding of Meis

Branchial arches develop following a first arch ground program, which is modified in Hox-positive segments (arches) to shape arch-specific morphologies (Minoux et al., 2009). The widespread and largely similar occupancy of Meis TFs in Hox-negative and Hox-positive branchial arches, and the observation that Meis TFs are essential for development of the entire branchial arch-derived skeleton (including the Hoxa2-positive IIBA), implicate Meis TFs in establishing the first arch ground state. We observed that the presence of Hoxa2 mainly induces quantitative, rather than qualitative, changes in Meis binding in the branchial arches (although the existence of a limited number of functional qualitative changes in Meis binding cannot be completely ruled out). By selectively reinforcing Meis binding, Hoxa2 appears to modulate the transcriptional program controlled by Meis in the branchial arches toward IIBA-specific transcription (Figure 7). Translating these genomic findings into

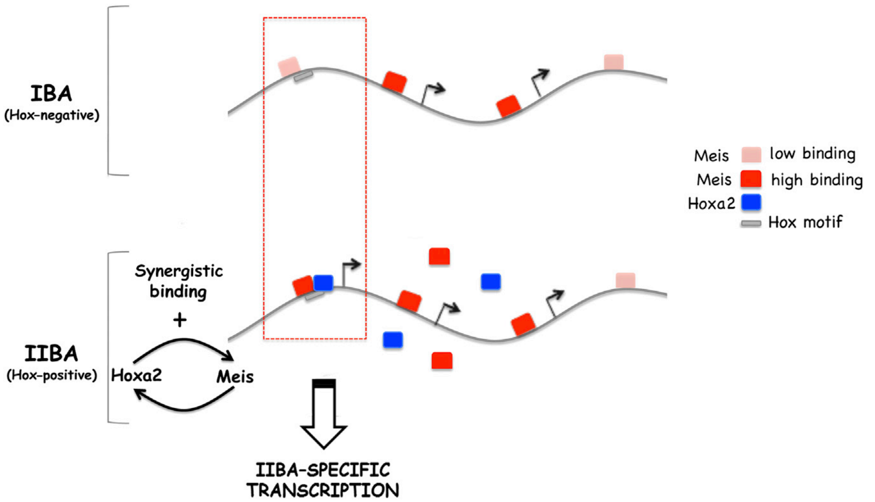

Figure 7. Synergistic Binding of Meis and Hoxa2 and IIBA-Specific Transcription

Schematic view of Meis occupancy on DNA in the Hox-negative (IBA) and Hox-positive tissues (IIBA). In the IBA, Meis TFs bind some DNA locations with low affinity (pink square) and some with high affinity (red square). In the IIBA, synergistic binding with Hoxa2 increases Meis-binding affinity at selected locations, which contains Hox recognition motifs (gray box, exemplified in red dashed rectangle). Additionally, a positive-feedback loop enhances Meis binding by increasing the levels of Hoxa2 and Meis in IIBA cells (blue and red squares, respectively). Enhanced binding of Meis is associated with genes highly expressed in the IIBA.

a genetic network is the requisite next step, and will require defining the functional contribution of individual nodes to the network. Indeed, Meis has a positive effect on Hox-dependent transcription (Choe et al., 2009, 2014), which is partly exerted by interfering with histone deacetylase recruitment by Pbx. In agreement, our results suggest that enhancement of Meis binding may turn poised enhancers into active ones.

Likewise, more posterior arches (third and fourth arches) share the first arch default state (Minoux et al., 2009). In contrast to their apparent lack of binding specificity, Hox proteins display multiple, paralog-specific Hox/TALE interaction modes (Hudry et al., 2012). These paralog-specific Hox/TALE interactions could affect the ground-state binding of Meis in different ways, thus creating the basis for instructing diverse, branchial arch-specific identities by Hox proteins from different paralog groups.

Our work shows that, molecularly, Hoxa2 does not entirely reprogram the epigenomic landscapes provided by Meis TFs in the branchial arches. Rather, Hoxa2 truly acts as a tissue-specific cofactor to specify the identity of the second arch, where it modulates the ground-state program established by Meis proteins. Our results provide a radically new framework to understand how Hox transcription factors control development in vertebrates.

EXPERIMENTAL PROCEDURES

Animal Experiments

Hoxa2 mutant mice were described previously (Gendron-Maguire et al., 1993; Rijli et al., 1993). CD1 mice were time-mated to isolate branchial arches from E11.5 embryos. Mouse experiments were carried out under ASPA 1986. Wild-type zebrafish were raised in the University of Massachusetts Medical Center Zebrafish Facility. In situ hybridization in mouse and zebrafish were carried out as described previously (Kanzler et al., 1998; Zannino and Appel, 2009).

Transfections

HEK293T cells were transfected using Fugene 6 (Promega), with a total of 1 μg DNA, containing 550 ng pGL3-Hoxa2 promoter (Hoxa2 promoter from -220 to +1 cloned in pGL3 [Promega]) and 150 ng of each Hoxa2, Meis1, and Pbx1a in pCDNA3 expression vector or pCDNA3 control (Life Technologies). Cells were harvested 24 hr after transfection for luciferase reporter assays (Promega). IBAs were dissociated into single cells and infected using supernatants from Ecotropic-Phoenix packaging cells, transfected with pMYs-IRES-GFP (Cell Biolabs) or pMYs-Hoxa2-IRES-GFP (Anderson et al., 2013). The infection efficiency, evaluated by fluorescence-activated cell sorting, was 70%. Cells were cultured for 72 hr and their chromatin extracted for chromatin immunoprecipitation (ChIP).

ChIP Assays and ChIP-Seq

ChIP-seq experiments have been deposited in ArrayExpress (Pbx IIBA, E-MTAB-1633; Meis IIBA, E-MTAB-1632; Meis IBA, E-MTAB-1631; and Foxc1 IIBA, E-MTAB-2696). ChIP-seq and ChIP assays were performed as described previously (Donaldson et al., 2012; Amin and Bobola, 2014) using the following antibodies: Meis1/2 (Santa Cruz Biotechnology sc-10599X), pan-Pbx (Santa Cruz Biotechnology, sc-25411X), H3K27ac (Abcam, ab4729), Hoxa2 (Kutejova et al., 2008), Foxc1 (Abcam, ab5079), and rabbit or goat immunoglobulin G (IgG) controls. Approximately 70 IBA pairs and 100 IIBA pairs were processed for each of the ChIP-seq experiments. Enrichment of IP material was validated by SYBR green quantitative PCR (qPCR) and percentage input was calculated for at least two duplicate samples. Primer sequences are listed in Table S5. ChIP was performed on zebrafish whole embryos (24 hours postfertilization) using an antibody that crossreacts with Meis1, Meis2, and Meis3 (Choe et al., 2009).

Bioinformatics Analysis

For ChIP-seq analysis, 50 bp sequences from Meis IBA ChIP, Meis IIBA ChIP, Pbx IIBA ChIP, Foxc1 ChIP-seq, and matched-input DNA controls were used. The Meis samples were run using two biological duplicates for the ChIP and matched-input DNA controls. Sequence reads were mapped to the NCBI37 (mm9/July 2007) release of the entire mouse (*Mus musculus*) genome using BFAST 0.7.0a (Homer et al., 2009a, 2009b). The mapped reads were converted into BED format for downstream analysis. Peak calling was performed using MACS version 1.4.2 (Zhang et al., 2008; http://liulab.dfci.harvard.edu/MACS/), using the matched-input DNA reads as a control. For peak calling, the "nomodel" parameter was used and the mean fragment size set at 200 bp. The threshold p value was set to $p < 1 \times 10^{-4}$. Binding regions with false discovery rate < 10% were selected (Meis IBA = 6,047, Meis IBA-rpt = 64,406, Meis IIBA = 17,676, Meis IIBA-rpt = 62,627, Pbx IIBA = 59,341, and Foxc1 IIBA = 30,834). The first replicate of Meis IBA and Meis IIBA underperformed, but 85.4% and 84.5% of the called regions were contained within the second replicates. In view of this, the second replicate was used in downstream analyses. The location of binding regions, defined by their summit region coordinates relative to RefSeq gene structure, was determined using CEAS version 0.9.9.8 (Shin et al., 2009; http://liulab.dfci.harvard.edu/CEAS/). The comparison of genome coordinates and the generation of the conservation profile used GALAXY (Goecks et al., 2010). Motif discovery and the scanning of known motifs in 200 bp summit regions and background sequences were described previously (Donaldson et al., 2012). The analysis of gene annotation enrichment was performed using GREAT version 2.0.2 (McLean et al., 2010; http://bejerano.stanford.edu/great/) using the "basal plus

extension" association rules. Plots and overlay density plots were generated using a python code available on request.

ACCESSION NUMBERS

The ArrayExpress accession numbers for the data sets reported in this paper are the following: Pbx IIBA, E-MTAB-1633; Meis IIBA, E-MTAB-1632; Meis IBA, E-MTAB-1631; and Foxc1 IIBA, E-MTAB-2696.

SUPPLEMENTAL INFORMATION

Supplemental Information includes Supplemental Experimental Procedures, five figures, and five tables and can be found with this article online at http://dx.doi.org/10.1016/j.devcel.2014.12.024.

AUTHOR CONTRIBUTIONS

S.A., D.Z., M.L., and F.L. carried out the experiments and analyzed the data. I.J.D, J.H., and M.R. performed computational analysis and interpretation of the data. N.B., C.S., F.S., S.A., and M.L. designed the experiment and analyzed and interpreted the data. N.B. wrote the manuscript. All authors read and edited the manuscript prior to submission.

ACKNOWLEDGMENTS

We thank Andy Sharrocks, Kim Mace, Moises Mallo, Karen Piper Hanley, Fabiana Luise, and Catherine Millar for critical reading of the manuscript and helpful discussions. We also thank the members of the Genomic Technologies and Bioinformatics Core Facilities at the University of Manchester, Dorothea Schulte and Carol Wicking, for the *Meis1* and *Zfp503* probes, respectively. This work was supported by the Biotechnology and Biological Sciences Research Council (BB/H018123/2 to M.R. and N.B.), the Medical Research Council (MR/L009986/1 to N.B.), the Wellcome Trust (097820/Z/11/Z to N.B.), and the NIH (NS038183 and HD065081 to C.S.), and facilitated by the Manchester Biomedical Research Centre.

Received: April 25, 2014
Revised: July 18, 2014
Accepted: December 23, 2014
Published: January 29, 2015

REFERENCES

Amin, S., and Bobola, N. (2014). Chromatin immunoprecipitation and chromatin immunoprecipitation with massively parallel sequencing on mouse embryonic tissue. Methods Mol. Biol. *1196*, 231–239.

Anderson, M., Amin, S., Luise, F., Zeef, L., and Bobola, N. (2013). Differential distribution of the Ca (2+) regulator Pcp4 in the branchial arches is regulated by Hoxa2. PLoS ONE *8*, e63160.

Azcoitia, V., Aracil, M., Martínez-A, C., and Torres, M. (2005). The homeodomain protein Meis1 is essential for definitive hematopoiesis and vascular patterning in the mouse embryo. Dev. Biol. *280*, 307–320.

Bardet, A.F., He, Q., Zeitlinger, J., and Stark, A. (2012). A computational pipeline for comparative ChIP-seq analyses. Nat. Protoc. *7*, 45–61.

Berger, M.F., Badis, G., Gehrke, A.R., Talukder, S., Philippakis, A.A., Peña-Castillo, L., Alleyne, T.M., Mnaimneh, S., Botvinnik, O.B., Chan, E.T., et al. (2008). Variation in homeodomain DNA binding revealed by high-resolution analysis of sequence preferences. Cell *133*, 1266–1276.

Biggin, M.D. (2011). Animal transcription networks as highly connected, quantitative continua. Dev. Cell *21*, 611–626.

Bobola, N., Carapuço, M., Ohnemus, S., Kanzler, B., Leibbrandt, A., Neubüser, A., Drouin, J., and Mallo, M. (2003). Mesenchymal patterning by Hoxa2 requires blocking Fgf-dependent activation of Ptx1. Development *130*, 3403–3414.

Carroll, S.B. (1995). Homeotic genes and the evolution of arthropods and chordates. Nature *376*, 479–485.

Chen, C.K., Symmons, O., Uslu, V.V., Tsujimura, T., Ruf, S., Smedley, D., and Spitz, F. (2013). TRACER: a resource to study the regulatory architecture of the mouse genome. BMC Genomics *14*, 215.

Choe, S.K., Lu, P., Nakamura, M., Lee, J., and Sagerström, C.G. (2009). Meis cofactors control HDAC and CBP accessibility at Hox-regulated promoters during zebrafish embryogenesis. Dev. Cell *17*, 561–567.

Choe, S.K., Ladam, F., and Sagerström, C.G. (2014). TALE factors poise promoters for activation by Hox proteins. Dev. Cell *28*, 203–211.

Colberg-Poley, A.M., Voss, S.D., Chowdhury, K., and Gruss, P. (1985). Structural analysis of murine genes containing homoeo box sequences and their expression in embryonal carcinoma cells. Nature *314*, 713–718.

Deflorian, G., Tiso, N., Ferretti, E., Meyer, D., Blasi, F., Bortolussi, M., and Argenton, F. (2004). Prep1.1 has essential genetic functions in hindbrain development and cranial neural crest cell differentiation. Development *131*, 613–627.

Dixon, J.R., Selvaraj, S., Yue, F., Kim, A., Li, Y., Shen, Y., Hu, M., Liu, J.S., and Ren, B. (2012). Topological domains in mammalian genomes identified by analysis of chromatin interactions. Nature *485*, 376–380.

Donaldson, I.J., Amin, S., Hensman, J.J., Kutejova, E., Rattray, M., Lawrence, N., Hayes, A., Ward, C.M., and Bobola, N. (2012). Genome-wide occupancy links Hoxa2 to Wnt-β-catenin signaling in mouse embryonic development. Nucleic Acids Res. *40*, 3990–4001.

Duboule, D., and Dollé, P. (1989). The structural and functional organization of the murine HOX gene family resembles that of Drosophila homeotic genes. EMBO J. *8*, 1497–1505.

Geisen, M.J., Di Meglio, T., Pasqualetti, M., Ducret, S., Brunet, J.F., Chedotal, A., and Rijli, F.M. (2008). Hox paralog group 2 genes control the migration of mouse pontine neurons through slit-robo signaling. PLoS Biol. *6*, e142.

Gendron-Maguire, M., Mallo, M., Zhang, M., and Gridley, T. (1993). Hoxa-2 mutant mice exhibit homeotic transformation of skeletal elements derived from cranial neural crest. Cell *75*, 1317–1331.

Goecks, J., Nekrutenko, A., and Taylor, J.; Galaxy Team (2010). Galaxy: a comprehensive approach for supporting accessible, reproducible, and transparent computational research in the life sciences. Genome Biol. *11*, R86.

Graham, A., Papalopulu, N., and Krumlauf, R. (1989). The murine and Drosophila homeobox gene complexes have common features of organization and expression. Cell *57*, 367–378.

Hisa, T., Spence, S.E., Rachel, R.A., Fujita, M., Nakamura, T., Ward, J.M., Devor-Henneman, D.E., Saiki, Y., Kutsuna, H., Tessarollo, L., et al. (2004). Hematopoietic, angiogenic and eye defects in Meis1 mutant animals. EMBO J. *23*, 450–459.

Homer, N., Merriman, B., and Nelson, S.F. (2009a). BFAST: an alignment tool for large scale genome resequencing. PLoS ONE *4*, e7767.

Homer, N., Merriman, B., and Nelson, S.F. (2009b). Local alignment of two-base encoded DNA sequence. BMC Bioinformatics *10*, 175.

Hudry, B., Remacle, S., Delfini, M.C., Rezsohazy, R., Graba, Y., and Merabet, S. (2012). Hox proteins display a common and ancestral ability to diversify their interaction mode with the PBC class cofactors. PLoS Biol. *10*, e1001351.

Hunt, P., Gulisano, M., Cook, M., Sham, M.H., Faiella, A., Wilkinson, D., Boncinelli, E., and Krumlauf, R. (1991). A distinct Hox code for the branchial region of the vertebrate head. Nature *353*, 861–864.

In der Rieden, P.M.J., Jansen, H.J., and Durston, A.J. (2011). XMeis3 is necessary for mesodermal Hox gene expression and function. PLoS ONE *6*, e18010.

Kanzler, B., Kuschert, S.J., Liu, Y.H., and Mallo, M. (1998). Hoxa-2 restricts the chondrogenic domain and inhibits bone formation during development of the branchial area. Development *125*, 2587–2597.

Kirilenko, P., He, G., Mankoo, B.S., Mallo, M., Jones, R., and Bobola, N. (2011). Transient activation of meox1 is an early component of the gene regulatory network downstream of hoxa2. Mol. Cell. Biol. *31*, 1301–1308.

Krumlauf, R. (1994). Hox genes in vertebrate development. Cell *78*, 191–201.

Kutejova, E., Engist, B., Mallo, M., Kanzler, B., and Bobola, N. (2005). Hoxa2 downregulates Six2 in the neural crest-derived mesenchyme. Development *132*, 469–478.

Kutejova, E., Engist, B., Self, M., Oliver, G., Kirilenko, P., and Bobola, N. (2008). Six2 functions redundantly immediately downstream of Hoxa2. Development *135*, 1463–1470.

Le Douarin, N.M., and Kalcheim, C. (1999). The Neural Crest: Developmental and Cell Biology Series, Second Edition. (Cambridge: Cambridge University Press).

Lemons, D., and McGinnis, W. (2006). Genomic evolution of Hox gene clusters. Science *313*, 1918–1922.

Longobardi, E., Penkov, D., Mateos, D., De Florian, G., Torres, M., and Blasi, F. (2014). Biochemistry of the tale transcription factors PREP, MEIS, and PBX in vertebrates. Dev. Dyn. *243*, 59–75.

Mann, R.S., Lelli, K.M., and Joshi, R. (2009). Hox specificity unique roles for cofactors and collaborators. Curr. Top. Dev. Biol. *88*, 63–101.

McGinnis, W., Garber, R.L., Wirz, J., Kuroiwa, A., and Gehring, W.J. (1984a). A homologous protein-coding sequence in Drosophila homeotic genes and its conservation in other metazoans. Cell *37*, 403–408.

McGinnis, W., Levine, M.S., Hafen, E., Kuroiwa, A., and Gehring, W.J. (1984b). A conserved DNA sequence in homoeotic genes of the Drosophila Antennapedia and bithorax complexes. Nature *308*, 428–433.

McLean, C.Y., Bristor, D., Hiller, M., Clarke, S.L., Schaar, B.T., Lowe, C.B., Wenger, A.M., and Bejerano, G. (2010). GREAT improves functional interpretation ofcis-regulatory regions. Nat. Biotechnol. *28*, 495–501.

Melvin, V.S., Feng, W., Hernandez-Lagunas, L., Artinger, K.B., and Williams, T. (2013). A morpholino-based screen to identify novel genes involved in craniofacial morphogenesis. Dev. Dyn. *242*, 817–831.

Minoux, M., Antonarakis, G.S., Kmita, M., Duboule, D., and Rijli, F.M. (2009). Rostral and caudal pharyngeal arches share a common neural crest ground pattern. Development *136*, 637–645.

Minoux, M., Kratochwil, C.F., Ducret, S., Amin, S., Kitazawa, T., Kurihara, H., Bobola, N., Vilain, N., and Rijli, F.M. (2013). Mouse Hoxa2 mutations provide a model for microtia and auricle duplication. Development *140*, 4386–4397.

Moens, C.B., and Selleri, L. (2006). Hox cofactors in vertebrate development. Dev. Biol. *291*, 193–206.

Nakamura, M., Runko, A.P., and Sagerström, C.G. (2004). A novel subfamily of zinc finger genes involved in embryonic development. J. Cell. Biochem. *93*, 887–895.

Nakamura, M., Choe, S.K., Runko, A.P., Gardner, P.D., and Sagerström, C.G. (2008). Nlz1/Znf703 acts as a repressor of transcription. BMC Dev. Biol. *8*, 108.

Nonchev, S., Vesque, C., Maconochie, M., Seitanidou, T., Ariza-McNaughton, L., Frain, M., Marshall, H., Sham, M.H., Krumlauf, R., and Charnay, P. (1996). Segmental expression of Hoxa-2 in the hindbrain is directly regulated by Krox-20. Development *122*, 543–554.

Noordermeer, D., and Duboule, D. (2013). Chromatin architectures and Hox gene collinearity. Curr. Top. Dev. Biol. *104*, 113–148.

Noyes, M.B., Christensen, R.G., Wakabayashi, A., Stormo, G.D., Brodsky, M.H., and Wolfe, S.A. (2008). Analysis of homeodomain specificities allows the family-wide prediction of preferred recognition sites. Cell *133*, 1277–1289.

Oulad-Abdelghani, M., Chazaud, C., Bouillet, P., Sapin, V., Chambon, P., and Dollé, P. (1997). Meis2, a novel mouse Pbx-related homeobox gene induced by retinoic acid during differentiation of P19 embryonal carcinoma cells. Dev. Dyn. *210*, 173–183.

Ovcharenko, I., Nobrega, M.A., Loots, G.G., and Stubbs, L. (2004). ECR Browser: a tool for visualizing and accessing data from comparisons of multiple vertebrate genomes. Nucleic Acids Res. *32*, W280–286.

Pearson, J.C., Lemons, D., and McGinnis, W. (2005). Modulating Hox gene functions during animal body patterning. Nat. Rev. Genet. *6*, 893–904.

Penkov, D., Mateos San Martín, D., Fernandez-Díaz, L.C., Rosselló, C.A., Torroja, C., Sánchez-Cabo, F., Warnatz, H.J., Sultan, M., Yaspo, M.L., Gabrieli, A., et al. (2013). Analysis of the DNA-binding profile and function of TALE homeoproteins reveals their specialization and specific interactions with Hox genes/proteins. Cell Rep. *3*, 1321–1333.

Polach, K.J., and Widom, J. (1996). A model for the cooperative binding of eukaryotic regulatory proteins to nucleosomal target sites. J. Mol. Biol. *258*, 800–812.

Rijli, F.M., Mark, M., Lakkaraju, S., Dierich, A., Dollé, P., and Chambon, P. (1993). A homeotic transformation is generated in the rostral branchial region of the head by disruption of Hoxa-2, which acts as a selector gene. Cell *75*, 1333–1349.

Runko, A.P., and Sagerström, C.G. (2003). Nlz belongs to a family of zinc-finger-containing repressors and controls segmental gene expression in the zebrafish hindbrain. Dev. Biol. *262*, 254–267.

Runko, A.P., and Sagerström, C.G. (2004). Isolation of nlz2 and characterization of essential domains in Nlz family proteins. J. Biol. Chem. *279*, 11917–11925.

Scott, M.P., and Weiner, A.J. (1984). Structural relationships among genes that control development: sequence homology between the Antennapedia, Ultrabithorax, and fushi tarazu loci of Drosophila. Proc. Natl. Acad. Sci. USA *81*, 4115–4119.

Selleri, L., Depew, M.J., Jacobs, Y., Chanda, S.K., Tsang, K.Y., Cheah, K.S., Rubenstein, J.L., O'Gorman, S., and Cleary, M.L. (2001). Requirement for Pbx1 in skeletal patterning and programming chondrocyte proliferation and differentiation. Development *128*, 3543–3557.

Shin, H., Liu, T., Manrai, A.K., and Liu, X.S. (2009). CEAS:cis-regulatory element annotation system. Bioinformatics *25*, 2605–2606.

Spitz, F., and Furlong, E.E. (2012). Transcription factors: from enhancer binding to developmental control. Nat. Rev. Genet. *13*, 613–626.

Thisse, C., and Thisse, B. (2005). High Throughput Expression Analysis of ZF-Models Consortium Clones. ZFIN Direct Data Submission. http://zfin.org/ZDB-PUB-051025-1.

Trainor, P.A., and Krumlauf, R. (2001). Hox genes, neural crest cells and branchial arch patterning. Curr. Opin. Cell Biol. *13*, 698–705.

Williams, T.M., Williams, M.E., and Innis, J.W. (2005). Range of HOX/TALE superclass associations and protein domain requirements for HOXA13:MEIS interaction. Dev. Biol. *277*, 457–471.

Yamaguchi, T.P., Bradley, A., McMahon, A.P., and Jones, S. (1999). A Wnt5a pathway underlies outgrowth of multiple structures in the vertebrate embryo. Development *126*, 1211–1223.

Yokoyama, S., Ito, Y., Ueno-Kudoh, H., Shimizu, H., Uchibe, K., Albini, S., Mitsuoka, K., Miyaki, S., Kiso, M., Nagai, A., et al. (2009). A systems approach reveals that the myogenesis genome network is regulated by the transcriptional repressor RP58. Dev. Cell *17*, 836–848.

Zannino, D.A., and Appel, B. (2009). Olig2+ precursors produce abducens motor neurons and oligodendrocytes in the zebrafish hindbrain. J. Neurosci. *29*, 2322–2333.

Zhang, Y., Liu, T., Meyer, C.A., Eeckhoute, J., Johnson, D.S., Bernstein, B.E., Nusbaum, C., Myers, R.M., Brown, M., Li, W., and Liu, X.S. (2008). Model-based analysis of ChIP-Seq (MACS). Genome Biol. *9*, R137.

Developmental Cell

Supplemental Information

Hoxa2 Selectively Enhances Meis Binding to Change a Branchial Arch Ground State

Shilu Amin, Ian J. Donaldson, Denise A. Zannino, James Hensman, Magnus Rattray, Marta Losa, François Spitz, Franck Ladam, Charles G. Sagerström, and Nicoletta Bobola

Supplemental Figures

Figure S1

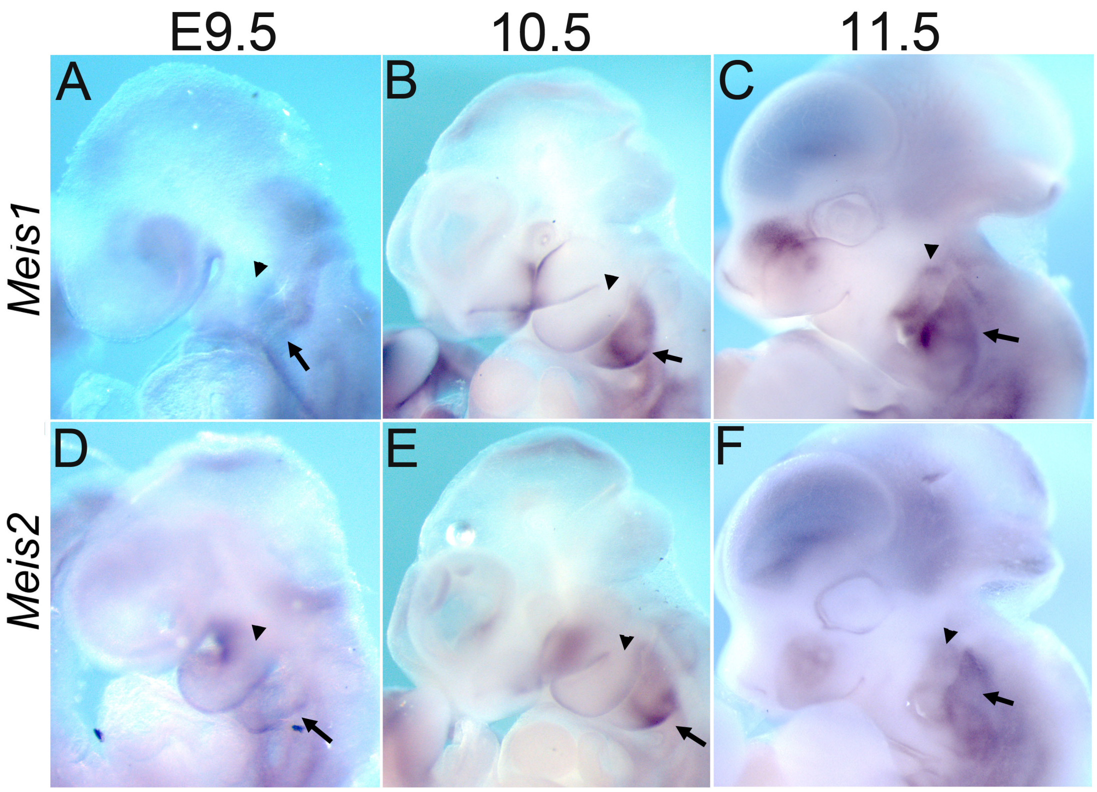

Figure S2

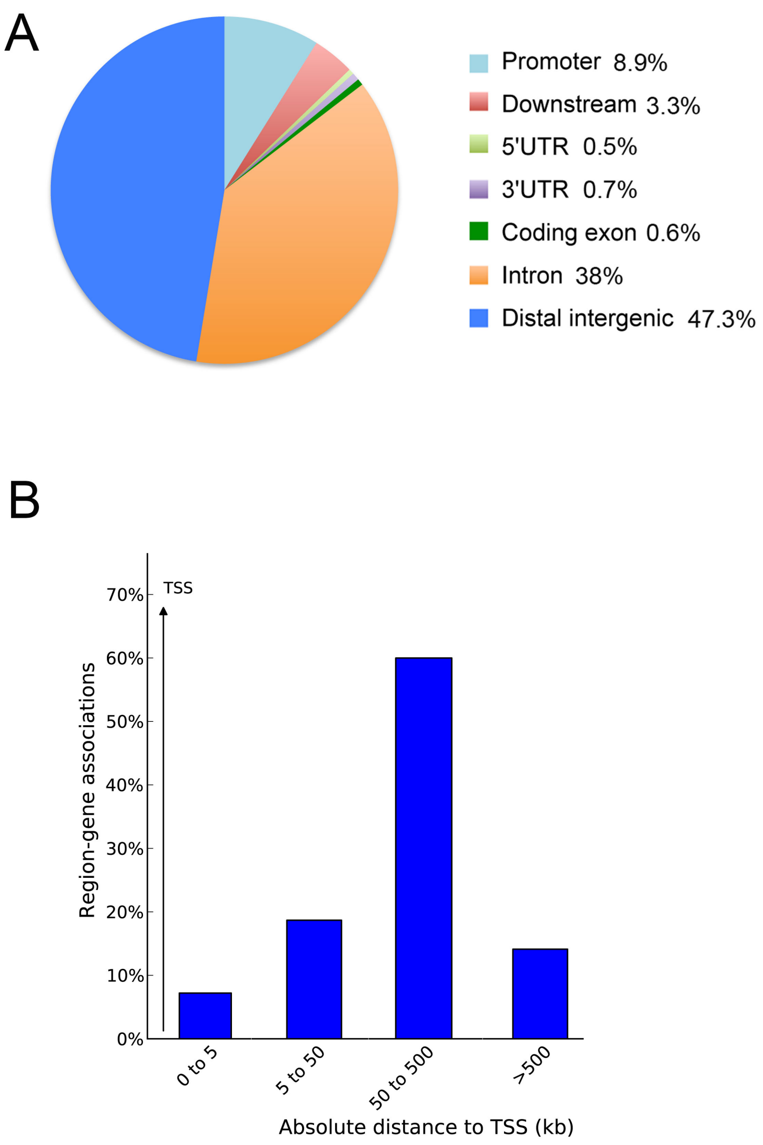
A
Promoter 8.9%
Downstream 3.3%
5'UTR 0.5%
3'UTR 0.7%
Coding exon 0.6%
Intron 38%
Distal intergenic 47.3%
B
TSS
70%
60%
50%
40%
30%
20%
10%
0%
Region-gene associations
0 to 5
5 to 50
50 to 500
>500
Absolute distance to TSS (kb)

Figure S3

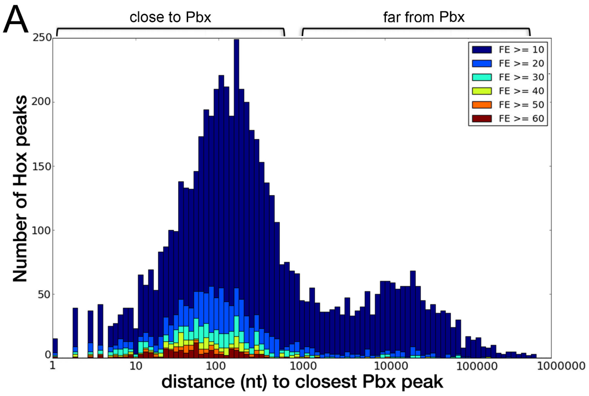

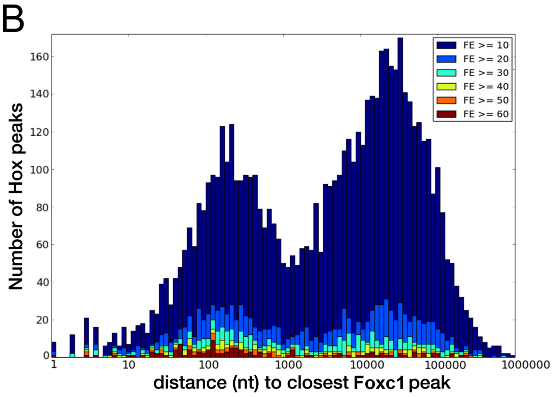

C

	Hoxa2	Meis	Pbx	Foxc1
Hoxa2	10031	5116	4688	1852
Meis		62627	31603	8015
Pbx			59341	7083
Foxc1				30384

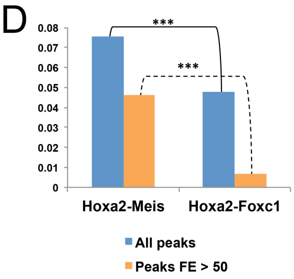

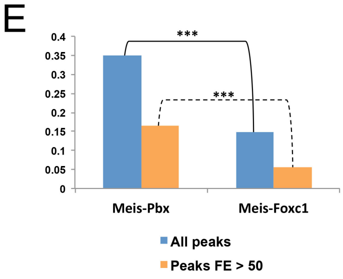

Figure S4

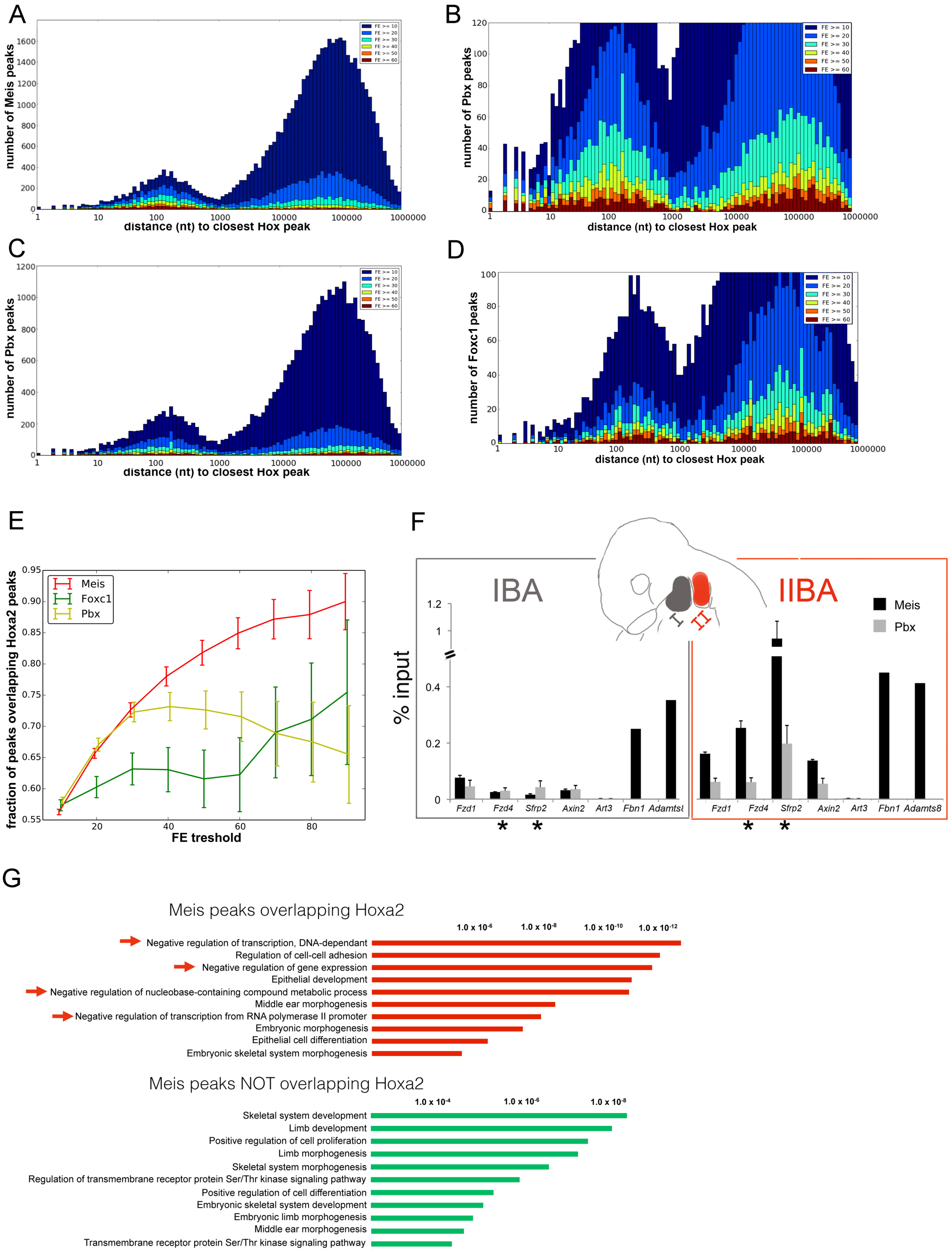
A
number of Meis peaks
distance (nt) to closest Hox peak
FE >= 10
FE >= 20
FE >= 30
FE >= 40
FE >= 50
FE >= 60
B
number of Pbx peaks
distance (nt) to closest Hox peak
C
number of Pbx peaks
distance (nt) to closest Hox peak
D
number of Foxc1 peaks
distance (nt) to closest Hox peak
E
fraction of peaks overlapping Hoxa2 peaks
FE treshold
Meis
Foxc1
Pbx
F
IBA
IIBA
% input
Meis
Pbx
Fzd1
Fzd4
Sfrp2
Axin2
Art3
Fbn1
Adamts8
I
II
G
Meis peaks overlapping Hoxa2
1.0 x 10-6
1.0 x 10-8
1.0 x 10-10
1.0 x 10-12
Negative regulation of transcription, DNA-dependant
Regulation of cell-cell adhesion
Negative regulation of gene expression
Epithelial development
Negative regulation of nucleobase-containing compound metabolic process
Middle ear morphogenesis
Negative regulation of transcription from RNA polymerase II promoter
Embryonic morphogenesis
Epithelial cell differentiation
Embryonic skeletal system morphogenesis
Meis peaks NOT overlapping Hoxa2
1.0 x 10-4
1.0 x 10-6
1.0 x 10-8
Skeletal system development
Limb development
Positive regulation of cell proliferation
Limb morphogenesis
Skeletal system morphogenesis
Regulation of transmembrane receptor protein Ser/Thr kinase signaling pathway
Positive regulation of cell differentiation
Embryonic skeletal system development
Embryonic limb morphogenesis
Middle ear morphogenesis
Transmembrane receptor protein Ser/Thr kinase signaling pathway

Figure S5

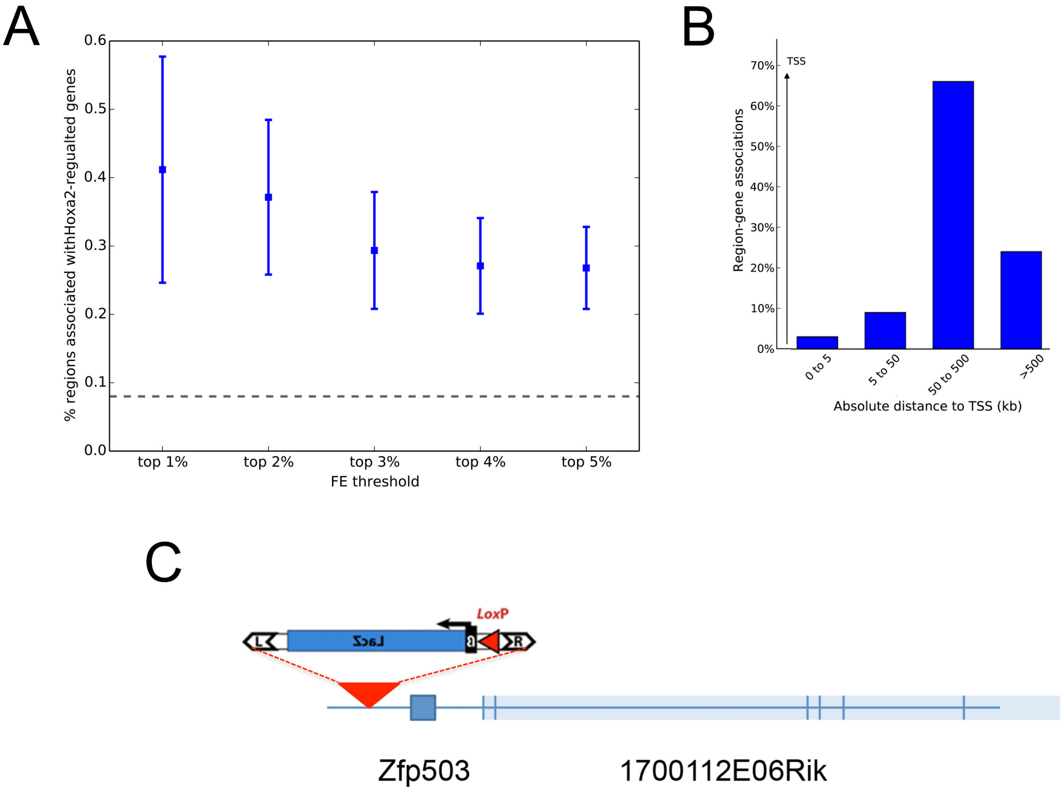

A
% regions associated withHoxa2-regualted genes
0.0
0.1
0.2
0.3
0.4
0.5
0.6
top 1%
top 2%
top 3%
top 4%
top 5%
FE threshold
B
Region-gene associations
TSS
0%
10%
20%
30%
40%
50%
60%
70%
0 to 5
5 to 50
50 to 500
>500
Absolute distance to TSS (kb)
C
LoxP
LacZ
L
R
Zfp503
1700112E06Rik

Supplemental Figures legends

Figure S1 (related to Figure 1). Expression of *Meis1* and *Meis2* in the craniofacial region. Whole-mount ISH on wild-type E9.5 (A, D), E10.5 (B, E) and late E11.5 (C, F) mouse embryos, using *Meis1* (A-C) and *Meis2* (D-F) probes. The IIBA (arrows) is one of the main domains of expression of *Meis1* and *Meis2*. Arrowheads indicate the first arch.

Figure S2 (related to Figure 2). **Genome-wide occupancy of Meis TFs in the IIBA**. Distribution of Meis summit regions relative to Reference Sequence (RefSeq) gene structures. The pie charts indicate the proportion of reads for each gene structure; the corresponding numeric values are included. B. Distribution of Meis summits relative to the absolute distance to a trancription start site (TSS).

Figure S3 (related to Figure 4). Combinatorial binding of Hoxa2, Meis, Pbx and Foxc1 A. Distance of Hoxa2 peaks (IIBA) relative to Pbx peaks. Hoxa2 peaks are binned according to distance to the nearest Pbx peak and labeled according to fold enrichment (FE; high FE is represented by dark red bars and low FE is represented by dark blue bars). B. Distance of Hoxa2 peaks relative to Foxc1 peaks. Hoxa2 peaks are binned according to distance to a nearest Foxc1 peak and labeled according to FE, as above. C. Total number of peaks for Hoxa2, Meis, Pbx and Foxc1 followed by pairwise combinations (number of overlapping 200nt summits). D, E. Significance of pairwise overlaps The co-binding frequency of Hoxa2/Meis is significantly higher than Hoxa2/Foxc1 (D). The co-binding frequency of Meis/Pbx is significantly higher than Meis/Foxc1 (E). Using a binomial test, similar results were observed using the entire ChIP-seq (blue bars) and peaks with FE>50 (orange bars). Asteriks indicate a P value <1E-8.

Figure S4 (related to Figure 5). Hoxa2 enhances Meis binding. A. Distance of Meis peaks (IIBA) relative to Hoxa2 peaks (non cropped). Meis peaks are binned according to distance to the nearest Hoxa2 peak and labeled according to fold enrichment (FE; high FE is represented by dark red bars and low FE is represented by dark blue bars). B, C. Distance of Pbx peaks relative to Hox peaks, cropped (B) and uncropped (C). Pbx peaks are binned according to distance to a nearest Hoxa2 peak and labeled according to FE, as above. D. Distance of Foxc1 peaks relative to Hox peaks (cropped). Foxc1 peaks are binned according to distance to a nearest Hoxa2 peak and labeled according to FE, as above. E. Distribution of Meis, Pbx and Foxc1 peaks relative to Hoxa2 binding. The fraction of Meis (red), Pbx (yellow) and Foxc1 (green) peaks close (1kb) to a Hoxa2 peak are plotted with error bars showing 95% confidence region. High Meis peaks (FE≥40) occur at significantly higher frequencies close to Hoxa2 (1kb) than Pbx or Foxc1 peaks. F. Meis and Pbx occupancy in the IBA (grey panel) and IIBA (red panel) by ChIP-qPCR. *Fzd1*, *Fzd4*, *Sfrp2* and *Axin2* are Hoxa2-bound regions; the IIBA/IBA average enrichment ratios at these regions were 10 and 2.41 for Meis and Pbx, respectively. Regions bound by Meis alone (i.e., *Fbn1, Adamts8*, closest Hoxa2 binding >10 kb) showed comparable enrichments when chromatin was extracted from the IBA or the IIBA. *Art3* is a negative control (unbound region). Asterisks indicate regions associated with Hoxa2-regulated genes. Data are presented as average of two independent experiments in duplicate and error bars represent the standard error of the mean. G. Functional categories associated with the top Meis peaks in the IIBA that overlap (red) or do not overlap (green) a Hoxa2 peak (200nt peak summit regions). Meis peaks overlapping Hoxa2 binding display enrichment in the GO term 'negative regulation of transcription' (red arrows), similar to the entire set of top Meis peaks in the IIBA. The length of the bars corresponds to the binomial raw (uncorrected) P-values (x-axis values).

Figure S5 (Related To Figure 6). Hoxa2 and Meis synergistic binding. A. Association of Hoxa2/Meis synergistic binding regions with Hoxa2-regulated genes. Increasing the number of 'synergistic binding events' (by relaxing the FE threshold) results in lower, but still significant associations with Hoxa2-regulated genes, relative to the entire Hoxa2 ChIP-seq (dashed horizontal line at 8%). Top1% (x-axis) indicates the set of top 1% Meis peaks that overlap top1% Hoxa2 peaks; top 2% indicates the set of top 2% Meis peaks that overlap top 2% Hoxa2 peaks, etc. The number of overlapping binding events are : top1%: n= 34; top 2%: n = 70; top 3%: n = 109; top 4%: n = 155; top 5%: n = 209. B. Distribution of Hoxa2/Meis synergistic binding regions relative to the transcription start site (TSS). C. Transposon insertion in the genomic regions containing *Zfp503*. The insertion is located downstream of *Zfp503* (red triangle).

Supplemental Tables and Legends

Table S1 (related to Figure 2). Meis ChIP-seq dataset in the IIBA

Table S2 (related to Figure 2). Meis ChIP-seq dataset in the IBA

Table S3 (related to Figure 2). Pbx ChIP-seq dataset in the IIBA

Table S4 (related to Figure 5). **Hoxa2-Meis synergistic binding regions.** List of high confidence Hoxa2-Meis cooperative binding, their associated genes and occurrence of Hox and Meis motifs. Genes dysregulated in *Hoxa2* mutant embryos are in bold.

Table S5 (related to Experimental procedure). List of primers used in ChIP-qPCR

**Gene**	**Forwards primer**	**Reverse primer**
Meis1 (1) enhancer	AGATGCCCAGAGAAAGCAAA	GGGGTGTGCATAGAAGGAAA
Meis1 (2) transcript	GCCAATCTTAGCGTCCATTT	TCACTCCGCCATTCTAAACA
Meis2 (1) enhancer	GGGGCGATGCTGTTTATTTA	GGGTGACAGGAATGATGGAT
Meis2 (2) promoter	TCCCGCAGACATCATTGTTA	AAATTTGGCCTCGTTCTCCT
Pou6f2 (Donaldson et al. 2012)	CCTGGATTTGACTCGGAAAG	CAGCTGTAACCCGACATTATGA
Itih4 neg (Donaldson et al. 2012)	GAGTCTGCTTGGCTTGAACC	AAGATTGGGCACTTTTTGGA
Hoxa2	TGAGGCGTTCCTTTCTGACT	GGGACCGCGCTACTATTAAA
Fzd1 (Donaldson et al. 2012)	GCCAGAAAATTGTTTGCATAAT	AGAGGGCTGTGGAATTCTGA
Fzd4 (Donaldson et al. 2012)	CTTCCTGGCTCTGCTGAAAG	CAAGGACTCCCATTTACCTCA
Sfrp2 (Donaldson et al. 2012)	TCACCATGACAGGTGGAAAA	CGGAGCTGAAACAGAAGCTC
Axin2 (Donaldson et al. 2012)	TGCTAAAAAGCCGGGAAGTA	TCGCAGTCCCTTTGAACTCT
Art3 neg (Donaldson et al. 2012)	GCCAAATTTAGCCAGATCCA	ACGGGGCAACAATTAGCTTT
Fbn1	CCTACCAGCCTGTGTCAATT	TCCTTTTCCCAAGTCTCCAGT
Adamts8	CAGCCTCCACTTAGCCAATT	GGCAACATTTCTGGCACTTTC

Zfp503	TCCTGGTCTGTTTAATGTTTGCT	GCCATCATCTAAAGCACAGAGG
Zfp703	GGCTCCGATGGCTGTAATAA	TGCATTTGCAAAGACGGCTA
Wnt5a	AAGACCTCCTTGCGATCTGA	AAACACCAGGGGCAATCATA
Cxcl12	CACTGATTGGAACCAGATGGG	GCTTGCCAAGTGTGTCGCTAA
Neg Chr17	TGAAGTACAGAAGCGAATGAAGG	TTTGCATGCCAGTGATACTTATC

Supplemental Experimental procedures

ISH probes

Probes used for *in situ* hybridization were *Meis1*, *Zfp503* (gifts from Dorothea Schulte and Carol Wicking), *Meis2* (targeting Meis2 3'UTR), *Zfp703* (Slorach et al., 2011) for mouse and *dlx2a* (Akimenko et al., 1994) and *hoxa2b* (Prince and Lumsden, 1994; Prince et al., 1998) for zebrafish.

Antisense morpholino oligonucleotides injections

Antisense morpholino (MO) oligonucleotides were designed to the translation start sites of *meis1* (5'- TATCTTCGTACCTCTGCGCCATCGC-3'), *meis2a* (5'-CTCATCGTACCTTTGCGCCATCAGC -3'), *meis3* (5'-AACTCCTCATACCTCTTATCCATGC-3'), and *meis4.1a* (5'-AGATCCTCGTACCGTTGCGCCATGA-3'), with a corresponding 5bp mismatch MO designed to *meis4.1a* (5'- AGtTgCTCcTACCcTTcCGCCATGA-3') (Gene Tools, LLC). A combination of four antisense morpholinos (MOs) was injected, each with a concentration of 0.7 mM (final combined concentration of 2.8 mM) in 1nl total volume. A corresponding 2.8 mM mismatch control MO was also used.
